# Seizure protein 6 and its homolog seizure 6-like protein are physiological substrates of BACE1 in neurons

**DOI:** 10.1186/s13024-016-0134-z

**Published:** 2016-10-05

**Authors:** Martina Pigoni, Johanna Wanngren, Peer-Hendrik Kuhn, Kathryn M. Munro, Jenny M. Gunnersen, Hiroshi Takeshima, Regina Feederle, Iryna Voytyuk, Bart De Strooper, Mikail D. Levasseur, Brian J. Hrupka, Stephan A. Müller, Stefan F. Lichtenthaler

**Affiliations:** 1 German Center for Neurodegenerative Diseases (DZNE), Munich, Germany; 2 Neuroproteomics, Klinikum rechts der Isar, Technische Universität München, Munich, Germany; 3 Institute for Advanced Study, Technische Universität München, Munich, Germany; 4 Institute for Pathology und Pathological Anatomy, Technische Universität München, Munich, Germany; 5 Department of Anatomy and Neuroscience, University of Melbourne, Victoria, Australia; 6 The Florey Institute of Neuroscience and Mental Health, University of Melbourne, Victoria, Australia; 7 Division of Pharmaceutical Sciences, Graduate School and Faculty of Pharmaceutical Sciences, Kyoto University, Kyoto, Japan; 8 Institute for Diabetes and Obesity, Monoclonal Antibody Research Group, Helmholtz Zentrum München, German Research Center for Environmental Health (GmbH), Munich, Germany; 9 VIB Center for the Biology of Disease, Leuven, Belgium; 10 Center for Human Genetics, and Leuven Institute for Neurodegenerative Diseases (LIND), University of Leuven (KU Leuven), Leuven, Belgium; 11 Institute of Neurology, University College London, London, UK; 12 Department of Neuroscience, Janssen Pharmaceutica NV, Beerse, Belgium; 13 Munich Cluster for Systems Neurology (SyNergy), Munich, Germany

**Keywords:** Alzheimer’s disease, BACE1, BACE2, Secretase, Neuroproteomics, Biomarker, SEZ6, SEZ6L

## Abstract

**Background:**

The protease BACE1 (beta-site APP cleaving enzyme) is a major drug target in Alzheimer’s disease. However, BACE1 therapeutic inhibition may cause unwanted adverse effects due to its additional functions in the nervous system, such as in myelination and neuronal connectivity. Additionally, recent proteomic studies investigating BACE1 inhibition in cell lines and cultured murine neurons identified a wider range of neuronal membrane proteins as potential BACE1 substrates, including seizure protein 6 (SEZ6) and its homolog SEZ6L.

**Methods and results:**

We generated antibodies against SEZ6 and SEZ6L and validated these proteins as BACE1 substrates in vitro and in vivo. Levels of the soluble, BACE1-cleaved ectodomain of both proteins (sSEZ6, sSEZ6L) were strongly reduced upon BACE1 inhibition in primary neurons and also in vivo in brains of BACE1-deficient mice. BACE1 inhibition increased neuronal surface levels of SEZ6 and SEZ6L as shown by cell surface biotinylation, demonstrating that BACE1 controls surface expression of both proteins. Moreover, mass spectrometric analysis revealed that the BACE1 cleavage site in SEZ6 is located in close proximity to the membrane, similar to the corresponding cleavage site in SEZ6L. Finally, an improved method was developed for the proteomic analysis of murine cerebrospinal fluid (CSF) and was applied to CSF from BACE-deficient mice. Hereby, SEZ6 and SEZ6L were validated as BACE1 substrates in vivo by strongly reduced levels in the CSF of BACE1-deficient mice.

**Conclusions:**

This study demonstrates that SEZ6 and SEZ6L are physiological BACE1 substrates in the murine brain and suggests that sSEZ6 and sSEZ6L levels in CSF are suitable markers to monitor BACE1 inhibition in mice.

**Electronic supplementary material:**

The online version of this article (doi:10.1186/s13024-016-0134-z) contains supplementary material, which is available to authorized users.

## Background

The β-secretase BACE1 (β-site APP cleaving enzyme) is a key drug target in Alzheimer’s disease (AD) [1]. BACE1 cleaves the amyloid precursor protein (APP) and thus catalyzes the first step in generation of the amyloid β peptide (Aβ) [2–5], which has a critical role in AD pathogenesis [6]. BACE1 is highly expressed in the nervous system and contributes to additional physiological processes besides its role in AD, e.g. through neuregulin-1 cleavage in myelination and CHL1 cleavage in axon targeting [7–12]. Moreover, several phenotypic changes were described in BACE1-/- mice, such as epileptic seizures, schizophrenic symptoms, increased mortality and altered insulin metabolism, but most of the BACE1 substrates contributing to these phenotypes still need to be determined [13]. Their identification and validation would also allow the estimation of potential liabilities of BACE inhibitors in AD clinical trials and the use of BACE1 substrate cleavage products, in addition to Aβ, as possible companion diagnostics to monitor BACE1 inhibition in animals and patients.

More than 40 substrate candidates for BACE1 were identified in recent proteomic studies in murine neurons or cerebrospinal fluid (CSF), but only a few of them have been validated to date with functional or in vitro assays, including L1, CHL1, ENPP5 and PTPRN2 [12, 14–16].

The three members of the seizure protein 6 (SEZ6) family, namely SEZ6, SEZ6-like (SEZ6L) and SEZ6-like 2 (SEZ6L2) have been identified as candidate BACE1 substrates in different studies [15, 17], but have not yet been validated in detail. The SEZ6 family controls synaptic connectivity and motor coordination in mice [18, 19], but little is known about the functions of these proteins at the molecular level. How BACE1-cleavage influences the function of SEZ6 and SEZ6L has not been investigated so far.

Interestingly, several of the identified BACE1 substrate candidates were also found to be cleaved by other proteases. As a result, substrate cleavage was only partly blocked upon BACE1 inhibition or BACE1-deficiency [14, 15], limiting the use of these substrates or their cleavage products as potential biomarkers to monitor BACE1 inhibition in vivo. In contrast, the two type I membrane proteins SEZ6 and its homolog SEZ6L appeared to be almost exclusively cleaved by BACE1 in neurons [15], making them potential biomarkers for BACE activity in vivo. The third family member, SEZ6L2, appeared to be mostly cleaved by proteases other than BACE1 [15, 17]. After the proteomic identification of SEZ6 as a BACE1 substrate candidate, SEZ6 was also shown to undergo reduced cleavage in BACE1-/- mouse brains [15]. However, the proteomic data for SEZ6L have not been validated by other methods and another proteomic study using pancreatic cells and tissue failed to confirm SEZ6L as a BACE1 substrate. Instead, that study demonstrated that SEZ6L is cleaved by the BACE1-homolog BACE2 in pancreas [17].

To resolve whether SEZ6 and SEZ6L are *bona fide* BACE1 substrates in brain, we generated monoclonal antibodies against both proteins and validated SEZ6 and SEZ6L as BACE1 substrates in murine neurons and brain. Additionally, SEZ6 and SEZ6L levels at the neuronal surface were controlled by BACE1, as demonstrated by cell surface biotinylation. Finally, we used a whole proteome analysis of CSF from BACE-deficient mice and found that the soluble ectodomains of SEZ6 and SEZ6L in CSF were most strongly reduced among all BACE1 substrates identified, suggesting their use as potential biomarkers in CSF to monitor BACE1 activity in mice.

## Methods

### Materials

The following antibodies were used: pAb SEZ6 [18], newly generated monoclonal SEZ6 and monoclonal SEZ6L (described below), pAb SEZ6L2 (R&D Systems, AF4916), pAb SEZ6L (R&D Systems, AF4804), 3D5 (kindly provided by Robert Vassar), pAb BACE2 (Santa Cruz, sc-10049), calnexin (Enzo, Stressgen, Farmingdale, NY, USA, ADI-SPA-860), β actin (Sigma, A5316), LDLR (R&D system, AF2255), rat mAb HA 3F10 (Roche, 11867423001), Flag M2 (Sigma, F1804), anti-DYKDDDDK (Biolegend, L5), anti-V5 (ThermoFisher, R960-25), HRP coupled anti-mouse and anti-rabbit secondary (DAKO), HRP coupled anti-goat, anti-rat and anti-sheep (Santa Cruz), biotinylated goat anti-rat IgG (Vector Laboratories), SULFO-TAG labelled anti-sheep (MSD, R32AI-1). The following reagents and media were used: neurobasal medium, HBSS and B27 (Invitrogen), C3 (β-secretase inhibitor IV; Calbiochem, 565788, final concentration 2 μM), DAPT (D5942 Sigma, final concentration 1 μM), ON-TARGETplus Bace2 siRNA SMARTpool, ON-TARGETplus Non-targeting Pool (Dharmacon, L-040326-00-0005 and D-001810-10-05, respectively), FlexiTube GeneSolution siRNA for Bace1 and AllStars Negative Control siRNA (Qiagen, GS23821 and SI03650318, respectively).

### Mouse strains

The following mice were used in this study: wild type (WT) C57BL/6NCrl (Charles River), BACE1-/- (Jackson Laboratory, strain B6.129- Bace1tm1Pcw/J, BACE1 KO), SEZ6-/- (SEZ6 KO) [18], SEZ6 family triple knockout (TKO) mice lacking SEZ6, SEZ6L and SEZ6L2 [19] and SEZ6L2-/- (SEZ6L2 KO, bred from SEZ6 family TKO [19]). For the CSF experiments the following mice were used: WT, single BACE1-/- (BACE1 KO), single BACE2-/- (BACE2 KO), double BACE1-/- BACE2-/- (BACE DKO) knockout mice [20]. All mice were on a C57BL/6 background and were maintained on a 12/12 h light-dark cycle with food and water *ad libitum*.

### Antibody production in rat

Monoclonal antibodies against murine SEZ6 (clone 14E5, IgG1) and murine SEZ6L (clone 21D9, IgG2a) were generated using standard procedures [21]. Briefly, a cDNA (HIS-mmSEZ6-HIS) was generated encoding murine (*mus musculus*) SEZ6 ectodomain (mmSEZ6, aa: 29-869, lacking the endogenous signal peptide) with an N- and C-terminal HIS tag, fused to an N-terminal CD5 signal peptide. The CD5 signal peptide allows for efficient secretion of the recombinant protein and is removed upon expression by signal peptidase, yielding HIS-mmSEZ6-HIS. The other cDNA (mmSEZ6L-1xStrepII) encoded murine SEZ6L ectodomain with its endogenous signal peptide (mmSEZ6L, aa: 1-812) and a C-terminal 1xStrepII tag. cDNA constructs were expressed in HEK293T cells and recombinant proteins were purified from the supernatant and used for immunization of rats.

### Immunohistochemistry

*DAB immunostaining:* Brains from 4 % paraformaldehyde perfusion-fixed SEZ6 TKO (*n* = 4) and WT (*n* = 7) adult mice were cryosectioned and underwent sequential incubation in BLOXALL (Vector Laboratories), 4 % Bovine Serum Albumin (BSA, Sigma Aldrich) and 0.1 % Triton X-100 (Sigma Aldrich) in phosphate buffered saline (PBS), and avidin/biotin (Avidin/Biotin Blocking Kit, Vector Laboratories). Sections were incubated overnight with monoclonal rat anti-SEZ6 or SEZ6L primary antibodies diluted in 2 % BSA and 0.3 % Triton X-100 in PBS. Sections were washed with PBS, incubated with biotinylated goat anti-rat IgG (Vector Laboratories) and processed using the VECTASTAIN ABC Kit (Vector Laboratories) and ImmPACT DAB peroxidase substrate as chromogen (Vector Laboratories) according to manufacturer’s instructions. Some sections were counterstained with haematoxylin. Primary or secondary antibodies were omitted on sections in each experiment to confirm staining specificity. Low power images were acquired on a Mirax slide scanner and high power images were acquired at 63× magnification on a Zeiss Axio microscope.

### Molecular biology

pcDNA3.1/HA-SLIC-Flag-mmSEZ6 was generated cloning full-length *Mus musculus* SEZ6, transcript variant 1 (Uniprot Q7TSK2-1) without signal peptide in pcDNA3.1 vector using Gibson assembly protocol as previously described [14]. The signal peptide of SEZ6 was replaced by the CD5 signal peptide, followed by a short tag resulting from sequence and ligase independent cloning (SLIC) [22], and an HA tag (YPYDVPDYA). A FLAG tag (DYKDDDDK) was cloned to the C terminus of the protein. pcDNA3.1/HA-SLIC-Flag-empty was used as control. pcDNA3.1/Flag-V5-hSEZ6-HA was generated cloning full-length *Homo sapiens* SEZ6, transcript variant 1 (Uniprot Q53EL9-1) into pcDNA3.1 vector. Following the endogenous signaling peptide, a Flag and V5 (PIPNPLLGLDST) tag were inserted, separated by a 10 amino acid glycine/serine linker sequence. An HA tag was cloned to the C terminus of the protein.

### Transfection and stable line generation

HEK293T stably expressing pcDNA3.1/HA-SLIC-Flag-mmSEZ6 or pcDNA3.1/HA- SLIC-Flag-empty as control were generated and cultured as previously described [14]. Cells were seeded in plates coated with Poly-D-lysine (Sigma, P6407). After 24 h medium was replaced with fresh medium supplemented with either C3, DAPT or DMSO as control. Collection of supernatants and cell lysates (described below) was done after 24 h. MIN6 were cultured in the same conditions, supplementing the medium with 2 mM L-glutamine and 50 μM β-mercaptoethanol (all from Invitrogen). Cells were transfected with 10 nM of BACE1, BACE2 and respective control siRNA using Lipofectamine RNAiMAX (Invitrogen, 13778-150), according to manufacturer’s instructions. Forty-eight hours post transfection, medium was replaced and cells were incubated for 24 h before collection of supernatants and cell lysis.

For drug inhibition studies, MIN6 cells were transfected with pcDNA3.1/Flag-V5-hSEZ6-HA as described above. Stable cell lines were generated using Geneticin (Gibco) selection pressure (800 μg/ml). MIN6 cells stably expressing Flag-V5-SEZ6 were seeded at a concentration of 300,000 cells/well in Falcon 24-well tissue culture plates (Corning, 353047). After 72 h, the medium was removed and replaced with fresh medium containing BACE inhibitors. Cells were treated with a nonselective BACE inhibitor (Compound A: (4aR,6R,8aS)-8a-(2,4-difluorophenyl)-6-(3-methylisoxazol-5-yl)-4a,5,6,8-tetrahydro-4H-pyrano[3,4-d] [1, 3] thiazin-2-amine [23], and 2 BACE1-selective inhibitors (Compound B: (5S)-2-amino-5-(2,6-diethyl-4-pyridyl)-3-methyl-5-(3-pyrimidin-5-ylphenyl)imidazol-4-one (AZD3839) [24] or Compound C: (5S)-2-amino-5-(2,6-diethyl-4-pyridyl)-3-methyl-5-(3-pyrimidin-5-ylphenyl)imidazol-4-one [25]. After 24 h of drug incubation, medium was removed, centrifuged to remove floating cells/cell debris (4000xg, 10 min), and analyzed for soluble shed Flag-V5-hSEZ6 as described below. For evaluation of endogenous SEZ6L shedding, wild-type MIN6 cells were seeded as above, and medium was replaced with drug-containing Opti-MEM (Gibco). After 24 h of drug exposure, Opti-MEM was removed and centrifuged to remove cell debris.

### Cellular Aβ assay

Cellular activity was assessed using the human SK-N-BE(2) neuroblastoma cell line expressing the wild-type amyloid precursor protein (hAPP695). BACE inhibitors described above were diluted and added to the cells, incubated for 18 h, and then measurements of Aβ42 were taken. Aβ42 was measured by a sandwich αlisa assay using biotinylated antibody (AbN/25) attached to streptavidin-coated beads and antibody (cAb42/26) conjugated acceptor beads. In the presence of Aβ42, the beads come into close proximity. The excitation of the donor beads provokes the release of singlet oxygen molecules that triggers a cascade of energy transfer in the acceptor beads, resulting in light emission. Aβ42 was quantified on an EnVision Multimode plate reader (Perkin Elmer) with excitation at 650 nm and emission at 615 nm.

### Enzymatic BACE1 and BACE2 assay

Primary BACE1 and BACE2 enzymatic activity was assessed by a FRET assay using an amyloid precursor protein (APP) derived 13 amino acids peptide contain the “Swedish” Lys-Met/Asn-Leu mutation of the APP β-secretase cleavage site as a substrate (Bachem, M-2465) and soluble BACE1(1 − 454) (Aurigene, Custom made) or soluble BACE2 (Enzo, BML-SE550). The APP peptide substrate (Mca-SEVNLDAEFRL(Dnp)RR-NH_2_) contains two fluorophores: 1) (7-methoxycoumarin-4-yl) acetic acid (Mca), a fluorescent donor with excitation wavelength at 320 nm and emission at 405 nm and, 2) 2,4-dinitrophenyl (Dnp), a proprietary quencher acceptor. An increase in fluorescence is linearly related to the rate of proteolysis. BACE1 or BACE2 were incubated with substrate and the inhibitor for 120 min in a 384-well plate. The amount of proteolysis is measured by fluorescence measurement in the Fluoroskan microplate fluorometer (Thermo Scientific). For the low control, no enzyme was added to the reaction mixture.

### Mesoscale (MSD) detection of sFlag-V5-SEZ6 and sSEZ6L

Detection of Flag-V5-SEZ6 and SEZ6L was done in Mesoscale Discovery MULTI-ARRAY 96-well plates (L15XA-3 or L15XB-3 respectively). sFlag-V5-SEZ6 was quantified using anti-DYKDDDDK Tag capture antibody (L5, Biolegend, 10 μg/ml), mouse monoclonal anti-V5 Epitope Tag detection antibody (R960-25, ThermoFisher, 1:20000 dilution) and SULFO-TAG labeled Protein A (1:4000 dilution) for anti-mouse quantification. SEZ6L was quantified by coating 30 μl of Opti-MEM medium diluted 1:25 in PBS to MSD High Bind plates overnight at 4 °C, followed by detection with 25 μl of R&D System anti-SEZ6L (AF4804, 2 μg/ml) and SULFO-TAG labeled Anti-Sheep antibody (MSD, R32AI-1, 1 μg/ml). For both assays, blocking and antibody dilutions were done in 0.1 % Blocker™ Casein (ThermoFisher) in PBS. Detection was done using 2× concentration of Read Buffer T (MSD, R92TC-1). Data were transformed to 0–100 % activity based on low controls (2.5 μM nonselective BACE inhibitor with nM potency) and high controls (0.02 % DMSO) within the same plate. IC50s were calculated in Graphpad Prism using the four parameter variable slope nonlinear fit model. All curves are based on biological replicates with at least two technical replicates.

### Isolation of primary neurons

Neurons from WT mice were isolated at E15/E16 and cultured as described previously [26]. After 5 days in vitro (DIV), neurons were washed with PBS and medium was replaced with fresh neurobasal supplemented with C3 or DMSO as control. After 48 h (7 DIV), supernatants from neurons were collected and cells were lysed.

### Cell lysate preparation

Supernatants from neurons, HEK293T and MIN6 cells were collected and cells were lysed as described previously [14]. Protein concentrations were quantified with an BCA assay (Uptima Interchim, UP95425) and 15–20 μg of total neuronal lysate, 8–10 μg of HEK293T lysate and 15–20 μg of MIN6 lysate were used for Western Blot analysis.

### Brain fractionation

Brains were isolated from P7 BACE1 KO mice and WT littermates. SEZ6 KO, SEZ6L2 KO and SEZ6 TKO and WT brains were collected from 4 to 5 month old male mice. All brains were processed as previously described [15]. Protein concentrations were quantified with an BCA assay (Uptima Interchim, UP95425) and 15–20 μg of total protein were used for Western Blot analysis.

### Murine CSF sampling

CSF was extracted from single BACE1 KO, BACE2 KO, BACE DKO mice and WT controls according to a previously described protocol [27]. CSF was put into a 0.5 ml LoBind tube (Eppendorf), centrifuged for 5 min at 800 × g, and transferred to a fresh tube and frozen at −80 °C. For mass spectrometric analysis 7 WT and 7 BACE DKO were sampled and 5 μl of each CSF sample was used. Immunoblots for the analysis of murine CSF were performed using 5 or 4 μl of CSF.

### Western blot analysis

Samples were boiled for 5 min at 95 °C in Laemmli buffer. For the detection of SEZ6L, Laemmli buffer without disulfide bridge reducing agents such as β-mercaptoethanol was used. Samples were separated on 8 % SDS-polyacrylamide gels. Schägger gels were used for the detection of C-terminal fragments (16.5 % separation gel, 10 % spacer gel [28]). PVDF membranes (Millipore) were incubated with primary antibody for 1–2 h at room temperature or at 4 °C overnight. After incubation with secondary antibody at room temperature for 1 h, membranes were developed with ECL prime (GE Healthcare, RPN2232V1).

### Deglycosylation assay

40 μg of neuronal lysate were treated with endoglycosidase H (Endo H, New England Biolabs, P0702), or Peptide-*N*-Glycosidase (PNGase F, New England Biolabs, P0704) according to the manufacturer’s protocol. For SEZ6L, non-reducing conditions were used (denaturation buffer was with 5 % SDS but no DTT). Afterwards, the samples were separated on 8 % SDS-polyacrylamide gel.

### Surface biotinylation

At 7 DIV, neurons were biotinylated with EZ-Link™ Sulfo-NHS-Biotin (ThermoFisher, 21217) according to manufacturer’s protocol. Quenching was done with ammonium chloride (50 mM) and BSA (1 %) in PBS and lysis with SDS lysis buffer (50 mM Tris-HCl pH 8, 150 mM NaCl, 2 mM EDTA, 1 % SDS). RIPA buffer (10 mM Tris-HCl pH 8, 150 mM NaCl, 2 mM EDTA, 1 % Triton, 0.1 % sodium deoxycholate, 0.1 % SDS) was used to dilute the samples. After sonication, protein concentrations were quantified and 80 μg of total lysate were incubated with 25 μl of High Capacity Streptavidin Agarose Resin (ThermoFisher, 20361), mixed overnight at 4 °C. Beads were washed in RIPA buffer and bound proteins were eluted by boiling at 95 °C in Laemmli buffer supplemented with 3 mM biotin. Eluted proteins were separated on 8 % SDS-polyacrylamide gel and Western blotting was performed.

### BACE1 in vitro digestion and mass spectrometric cleavage site determination

The murine SEZ6 peptide AASLDGFYNGRSLDVAKAPAASSAL (PSL Peptide Specialty Laboratories GmbH, Germany) was resuspended in LC-MS grade water (Chromasolv, Sigma Aldrich, Germany) and 40 μg of peptide were used to determine the cleavage site. Peptides were incubated with recombinant BACE1 with or without C3 inhibitor in 50 mM sodium acetate buffer pH 4.4 from 4 to 16 h as previously described [29].

Samples from the peptide cleavage assay were analyzed by LC-MS/MS. An amount of 500 fmol with respect to the starting material of the synthetic peptide was injected. Samples were separated on a nanoLC system (EASY-nLC 1000, Proxeon – part of Thermo Scientific, US; PRSO-V1 column oven: Sonation, Germany) using an in-house packed C18 column (30 cm × 75 μm ID, ReproSil-Pur 120 C18-AQ, 1.9 μm, Dr. Maisch GmbH, Germany) with a binary gradient of water (A) and acetonitrile (B) containing 0.1 % formic acid at 50 °C column temperature and a flow of 250 nl/min (0 min, 8 % B; 25:00 min, 35 % B; 30:00 min, 95 % B; 40:00 min, 95 % B). The nanoLC was coupled online via a nanospray flex ion source (Proxeon – part of Thermo Scientific, US) to a Q-Exactive mass spectrometer (Thermo Scientific, US). The five most intense ions exceeding an intensity of 1.0 × 10^4^ were chosen for collision induced dissociation. The dynamic exclusion was reduced to 1 s and the m/z values of the proposed cleavage products were put on an inclusion list to get high quality MS/MS spectra.

MS raw data of the peptide cleavage assay were used to check for m/z values of possible cleavage products. Quantification was done by calculating the area under the curve of cleavage products using extracted ion chromatograms. Peak areas of the synthetic peptide incubated with BACE1 were compared with the control incubations of BACE1 and C3 as well as without BACE1. The identity of cleavage products was verified by a database search against the sequence of the synthetic peptide with Maxquant [30]. Non-specific cleavage was applied to identify cleavage products by tandem MS spectra.

### Mass spectrometric analysis of CSF samples

Seven WT and seven BACE DKO CSF samples were used for mass spectrometric analysis. A volume of 5 μL of CSF per sample was subjected to proteolytic digestion in 50 mM ammonium bicarbonate with 0.1 % sodium deoxycholate (Sigma Aldrich, Germany). Disulfide bonds were reduced by addition of 2 μL 10 mM dithiothreitol (Biomol, Germany). Cysteine residues were alkylated by addition of 2 μL 55 mM iodoacetamide (Sigma Aldrich, Germany). Proteolytic digestion was performed by consecutive digestion with LysC (0.1 μg; 4 h) and trypsin (0.1 μg; 16 h) at room temperature (Promega, Germany).

Samples were acidified by adding 4 μL of 8 % formic acid (Sigma Aldrich, Germany) and 150 μL of 0.1 % formic acid (Sigma Aldrich Germany). Precipitated deoxycholate was removed by centrifugation at 16,000 g for 10 min at 20 °C. Proteolytic peptides were desalted by stop and go extraction (STAGE) with C18 tips [31], dried by vacuum and dissolved in 20 μL 0.1 % formic acid.

Samples were analyzed with the same LC-MS/MS method as described for the BACE1 in vitro digestion assay with a longer gradient (0 min, 2 % B; 3:30 min, 5 % B; 137:30 min, 25 % B; 168:30 min, 35 % B; 182:30 min, 60 % B; 185 min, 95 % B; 200 min, 95 % B).

Full MS spectra were acquired at a resolution of 70,000. The top ten peptide ions exceeding an intensity of 1.5 × 10^4^ were chosen for collision induced dissociation. Fragment ion spectra were acquired at a resolution of 17,500. A dynamic exclusion of 120 s was used for peptide fragmentation.

### MS data analysis of CSF samples

The data were analyzed with Maxquant software (maxquant.org, Max-Planck Institute Munich) version 1.5.3.12 [30]. The MS data were searched against a reviewed canonical fasta database of *Mus musculus* from UniProt (download: January 26th 2016, 16758 entries). Trypsin was defined as protease. Two missed cleavages were allowed for the database search. The option first search was used to recalibrate the peptide masses within a window of 20 ppm. For the main search, peptide and peptide fragment mass tolerances were set to 4.5 and 20 ppm, respectively. Carbamidomethylation of cysteine was defined as static modification. Acetylation of the protein N-term as well as oxidation of methionine were set as variable modifications. False discovery rate for both peptides and proteins was adjusted to less than 1 % using a target and decoy approach (concatenated forward/reverse database). Only unique peptides were used for quantification. Label-free quantification (LFQ) of proteins required at least two ratio counts of unique peptides.

The LFQ intensity values were log2 transformed and a two-sided Welch’s t-test was used to evaluate the significance of proteins with changed abundance between KO and WT animals. A *p*-value less than 5 % was set as significance threshold.

### Statistical tests

Statistical differences for Western Blot experiments were determined using two-tailed Mann-Whitney test (GraphPad Prism Software, San Diego, CA, USA). In Fig. 7, one-way ANOVA followed by two-tailed Student’s t-Test, was used for Western Blot quantification. Graphs show mean ± SEM.

## Results

### Validation of new monoclonal antibodies against SEZ6 and SEZ6L

To validate SEZ6 and SEZ6L as BACE1 substrates, rat monoclonal antibodies against both proteins were generated. They were first tested in immunoblots using membrane fractions from mouse brains. As a control, the third family member, SEZ6L2, was also analyzed, using a commercial antibody. To ensure the specificity of the immunoblot signals, brains from wild type (WT) as well as from SEZ6-/- (SEZ6 KO) or SEZ6L2-/- (SEZ6L2 KO) mice were used. As SEZ6L-/- mouse brains were not available, brains from mice lacking all three SEZ6 family members (SEZ6-/-, SEZ6L-/-, SEZ6L2-/-; triple knock-out, TKO [16]) were used instead.

**Fig. 1:**
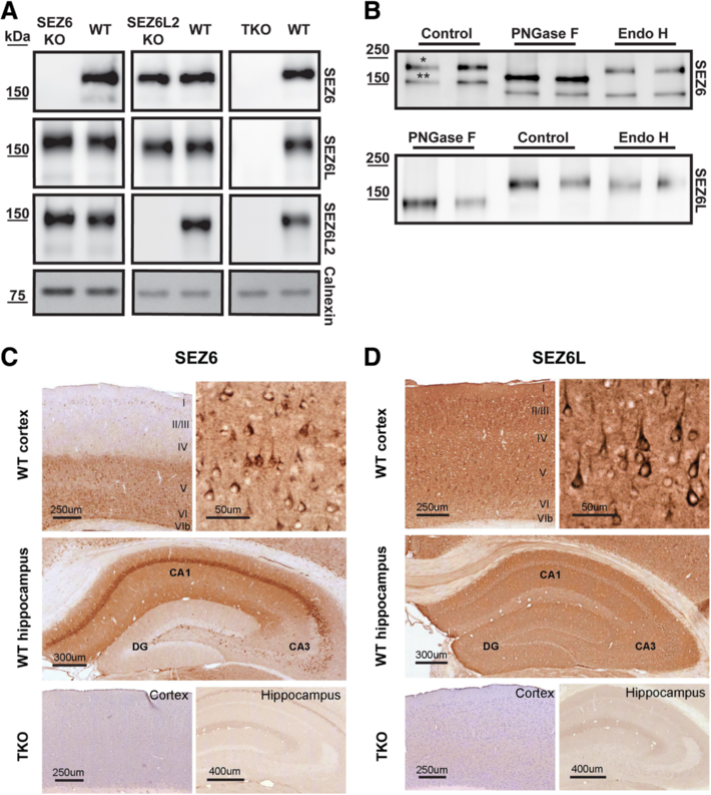
Specificity of SEZ6 and SEZ6L monoclonal antibodies. **a** Membranes from mouse brains were probed with the indicated antibodies against SEZ6, SEZ6L, SEZ6L2 or calnexin. Brains were collected from wild type (WT), SEZ6-/- (SEZ6 KO), SEZ6L2-/- (SEZ6L2 KO) or triple knock-out (TKO) mice lacking SEZ6, SEZ6L and SEZ6L2. **b** Lysates from primary neurons were treated with peptide N-glycosidase F (PNGaseF) or endoglycosidase H (EndoH) and blotted for SEZ6 and SEZ6L. For SEZ6, a polyclonal antibody was used in the deglycosylation experiment. * indicates mature SEZ6, ** indicates immature SEZ6. **c**, **d** Immunohistochemistry of TKO and WT brains using antibody against SEZ6 (**c**) or SEZ6L (**d**)

In WT brains the SEZ6 antibody detected a major band at 170 kDa and a band of minor intensity at 150 kDa (Fig. 1a). Importantly, both bands were absent in SEZ6 KO and TKO brains, but were clearly visible in SEZ6L2 KO brains, demonstrating the specificity of the SEZ6 antibody. Because SEZ6 has 10 predicted N-glycosylation sites [32], we next determined whether the two SEZ6 bands differ in their extent of glycosylation. In order to detect both the major and the minor band more intensively, a SEZ6 polyclonal antibody was used. Endogenous SEZ6 from neuronal lysates was deglycosylated in vitro using peptide N-glycosidase F (PNGaseF), which removes all N-linked sugars, or endoglycosidase H (EndoH), which only removes high-mannose sugars but not complex glycosylated sugars. PNGaseF induced a band shift and lowered the apparent molecular weight of both SEZ6 bands to 155 and 135 kDa, respectively (Fig. 1b). This demonstrates that SEZ6 is N-glycosylated. However, the fact that still two distinct SEZ6 bands – and not just one - were visible demonstrates that both protein forms must differ by an additional post-translational modification other than N-glycosylation. This is likely to be O-glycosylation as SEZ6 was found to be O-glycosylated in a proteomic study identifying O-glycosylated proteins [33]. Similar to PNGaseF, EndoH induced a band shift of the 150 kDa band, but did not induce a major shift of the 170 kDa band (Fig. 1b). This reveals that the 170 kDa band contains complex sugars (referred to as mature SEZ6), whereas the 150 kDa band (referred to as immature SEZ6), contains only high-mannose sugars.

The SEZ6L antibody detected one major band at 160 kDa and a very weak band at 130 kDa (Fig. 1a). Both bands were not detected in the SEZ6 TKO samples, while they showed unchanged intensity in WT, SEZ6 KO and SEZ6L2 KO brains, thus confirming the specificity of the antibody for SEZ6L. Similar to SEZ6, the major SEZ6L band at 160 kDa was complex N-glycosylated. The glycosylation was removed with PNGaseF, but not with EndoH (Fig. 1b). The 130 kDa band of SEZ6L was not consistently detected in the deglycosylation experiments, but may represent the immature form, similar to SEZ6.

As a control, SEZ6L2 expression was detected in WT and SEZ6 KO brains, but was absent in SEZ6L2 KO and SEZ6 TKO brains (Fig. 1a). Notably, in brains deficient in SEZ6 or SEZ6L2, levels of the other family members were not significantly altered (Fig. 1a), revealing that there are no compensatory changes in protein levels at least for deficiency of SEZ6 and SEZ6L2.

Taken together, these results demonstrate that SEZ6 and SEZ6L are N-glycosylated proteins and that the newly generated antibodies specifically detect endogenous SEZ6 and SEZ6L.

In WT adult mouse brains SEZ6 protein was localized to a number of brain regions including the neocortex and hippocampus (Fig. 1c), with particularly strong immunoreactivity in the striatum and olfactory tubercle (not shown). In the cortex SEZ6 was localized to neuronal cell bodies and processes, predominantly in layers V and VI (Fig. 1c). In the hippocampus, SEZ6 was localized to CA1 pyramidal neuron cell bodies and dendrites, CA2 and a subset of CA3 neurons, and sparsely labeled neurons in the dentate gyrus which resemble interneurons.

SEZ6 immunostaining was completely absent in SEZ6 TKO brain sections (Fig. 1c) and in SEZ6 KO brain sections (data not shown).

Similarly, SEZ6L immunoreactivity (Fig. 1d) appeared strong in the neocortex and hippocampus, and protein localization in these areas was consistent with SEZ6L mRNA expression in the Allen Mouse Brain Atlas [34]. SEZ6L localized to pyramidal neurons throughout the cortex, particularly the apical dendrites (Fig. 1d), and appeared relatively lower in layer IV and VI. All regions of the hippocampus displayed immunoreactivity for SEZ6L (Fig. 1d) although staining was less prominent in neuronal soma than the SEZ6 staining (Fig. 1c). SEZ6L staining was observed in other brain regions including the cerebellum and septal nuclei (data not shown). SEZL6 immunostaining was completely absent in SEZ6 TKO brain sections (Fig. 1d).

Taken together, the newly generated antibodies specifically detect endogenous SEZ6 and SEZ6L by immunohistochemistry as well as Western Blot.

### BACE1 cleavage of SEZ6 and SEZ6L in primary neurons and mouse brain

**Fig. 2:**
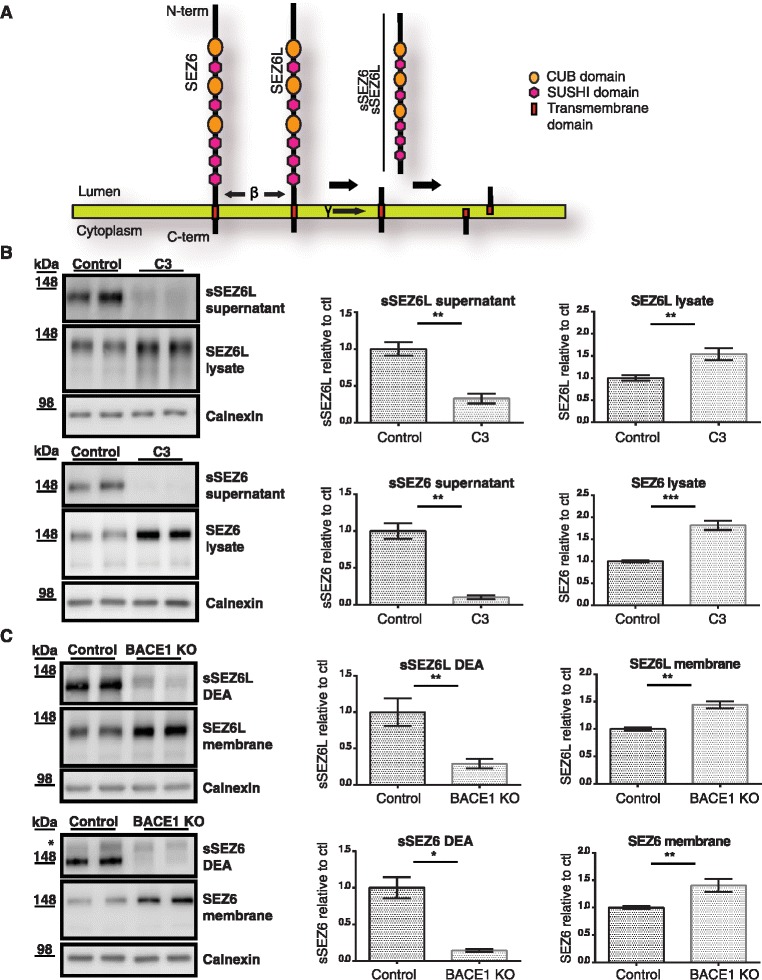
BACE1 is required for SEZ6 and SEZ6L shedding in primary neurons and mouse brain. **a** Schematic diagram of SEZ6 and SEZ6L domain structure and proposed proteolytic processing. **b** Detection of soluble SEZ6 and SEZ6L ectodomains (sSEZ6 and sSEZ6L) and full-length SEZ6 and SEZ6L in neuronal supernatant and lysate upon C3 treatment. **c** Detection of sSEZ6 and sSEZ6L and full-length SEZ6 and SEZ6L in BACE1 KO and WT brains. Brains were separated into soluble fraction (DEA) and membranes (membrane). Note that in this figure, a different molecular weight marker has been used compared to Fig. 1. The 148 kDa band corresponds to the band detected at 170 kDa in Fig. 1. The upper band in panel 2C (*) is due to unspecific signal. Densitometric quantitations of the Western blots are shown, (*; *p* < 0.05, **; *p* < 0.01, two-tailed Mann-Whitney test *n* = 6)

As a result of BACE1 cleavage, the soluble ectodomains of SEZ6 and SEZ6L (sSEZ6 and sSEZ6L) should be shed into the conditioned medium of primary neurons and into the extracellular space in mouse brains (Fig. 2a). However, when BACE1 is inhibited or deleted, sSEZ6 and sSEZ6L might be absent or strongly reduced. In fact, treatment of primary neurons with the established BACE1 inhibitor C3 (also known as BACE1 inhibitor IV) [35] strongly reduced sSEZ6L levels compared to the control treatment with a concomitant moderate increase of full-length SEZ6L levels in the cell lysate (Fig. 2b). Likewise, in P7 BACE1 KO mouse brains sSEZ6L was strongly reduced in the diethylamine soluble DEA brain fraction, while full-length SEZ6L was increased in the membrane fraction (Fig. 2c). In agreement with our previous study on SEZ6 [15], similar results were obtained for sSEZ6 and full-length SEZ6 both in C3-treated neurons and in BACE1 KO mouse brains (Fig. 2b and c). Taken together, these results reveal that ectodomain shedding of sSEZ6 and sSEZ6L requires BACE1 activity both in primary neurons and in mouse brains.

### BACE1 cleavage of SEZ6 and SEZ6L in pancreatic MIN6 cells

**Fig. 3:**
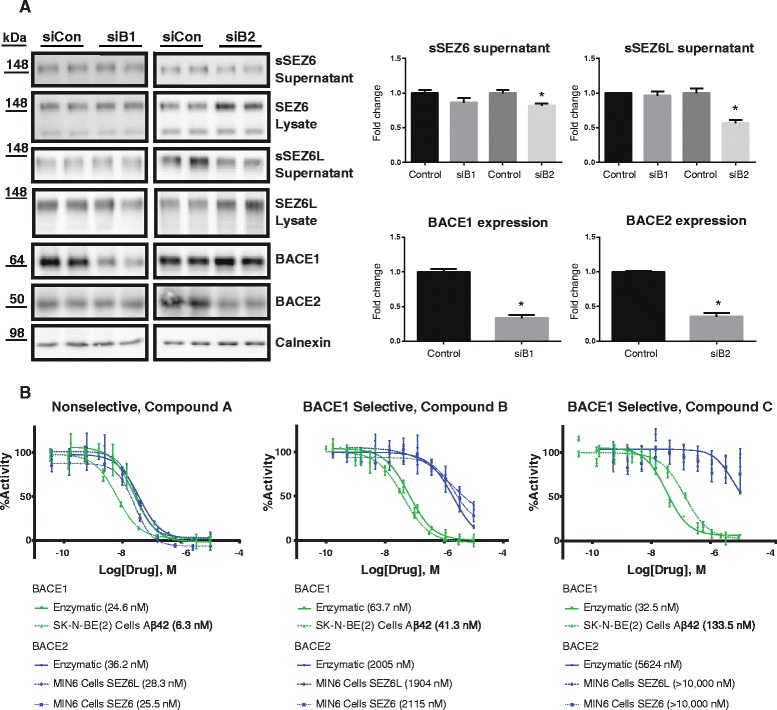
BACE2 but not BACE1 cleaves SEZ6 and SEZ6L in a pancreatic β-cell line. **a** sSEZ6 and sSEZ6L were detected in the supernatant and full-length SEZ6 and SEZ6L in the lysate of the pancreatic β-cell line MIN6 upon BACE1 and BACE2 knock-down by siRNA (siB1, siB2). As a control, cells were treated with non-silencing control siRNA (siCon). Densitometric quantitations of the Western blots are shown, (*; *p* < 0.05, two-tailed Mann-Whitney test *n* = 4). **b** BACE1 (*green lines*) and BACE2 (*blue lines*) activity were quantified in enzymatic (*solid lines*) and cellular (*dotted lines*) models after pharmacological inhibition with nonselective (inhibiting both BACE1 and BACE2, compounds B and C) and BACE1-selective inhibitors (compound A). Soluble Aβ42 as well as sSEZ6 and sSEZ6L were detected in the supernatant of the neuroblastoma cell line SK-N-BE(2) or in the MIN6 respectively, as indicated. Data were standardized to low and high controls within each assay. Data represent biological duplicates with two or more technical replicates

A previous proteomic study showed that SEZ6L was cleaved by BACE2, but not by BACE1 in the pancreatic β-cell line MIN6 [17], which is different from our findings in neurons and brain. SEZ6 was not detected in that study. To investigate whether the same differences can be observed for SEZ6, we used the same cell line MIN6 and knocked-down BACE1 or BACE2 with siRNAs (Fig. 3a). As a control, cleavage of SEZ6L was also monitored. In agreement with the previous study [17], sSEZ6L was reduced upon knock-down of BACE2, but not of BACE1. Interestingly, sSEZ6 was also not reduced upon knock-down of BACE1, but mildly reduced upon knock-down of BACE2. This shows that both SEZ6 and SEZ6L are not substrates for BACE1 in the pancreatic cell line. Full-length SEZ6 and SEZ6L levels were increased upon BACE2 knock-down, in line with the reduced cleavage of both proteins (Fig. 3a). Taken together, this demonstrates that both SEZ6 and SEZ6L are cleaved by different proteases in a tissue-specific manner. One possible scenario might be that the tissue-specificity reflects the relative amounts of BACE1 and BACE2 in different tissues. For example, BACE1 – which was the major SEZ6 and SEZ6L protease in neurons – was found to be expressed at higher levels in neurons compared to MIN6 cells (Additional file 1: Figure S1). The opposite was seen for BACE2, which was the primary protease cleaving SEZ6 and SEZ6L in MIN6 cells. This tissue- specificity is reminiscent of two other BACE1 substrates, APP and L1, which are mostly cleaved by BACE1 in neurons, but by ADAM10 in non-neuronal cells [15, 36–39].

The cleavage of SEZ6 and SEZ6L in MIN6 cells by BACE2, but not BACE1, was further evaluated using nonselective (inhibiting both BACE1 and BACE2) and BACE1-selective pharmacological inhibitors by assessing shedding of SEZ6 and SEZ6L in MIN6 cells. Because SEZ6 is expressed at low levels in MIN6 cells (Additional file 1: Figure S1), human SEZ6 tagged with an N-terminal Flag- and V5-tag (Flag-V6-hSEZ6) was mildly overexpressed in MIN6 cells. To validate the efficacy of BACE1 inhibition, Aβ42 (a BACE cleavage product of APP) was measured in the SK-N-BE(2) neuroblastoma cell model, and sFlag-V5-hSEZ6 and endogenous sSEZ6L in MIN6 cells. IC50s for the released substrate cleavage products (sFlag-V5-SEZ6 sSEZ6L) were compared with IC50s determined in enzymatic BACE1 and BACE2 assays. Cleavage of Aβ42 and sFlag-V5-SEZ6 and sSEZ6L were similar after addition of nonselective BACE inhibitor A and was consistent with equipotent inhibition of BACE1 and BACE2 in enzymatic assays. However, cleavage of sFlag-V5-SEZ6 and sSEZ6L was less impacted than Aβ42 upon inhibition with BACE1-selective inhibitors (B and C) and followed the enzymatic inhibition curves of BACE2 rather than BACE1 (Fig. 3b). This confirms the findings in Fig. 3a and demonstrates that in MIN6 cells SEZ6 and SEZ6L are predominantly cleaved by BACE2, but not by BACE1.

### BACE1 inhibition increases neuronal cell surface levels of SEZ6 and SEZ6L

**Fig. 4:**
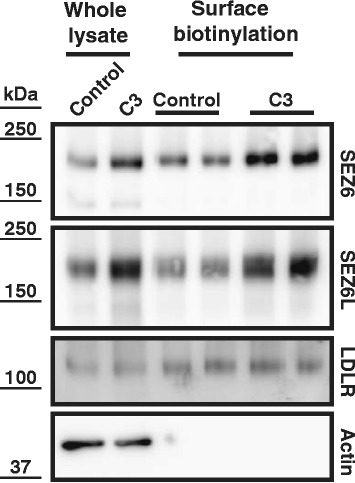
BACE1 controls neuronal cell surface levels of SEZ6 and SEZ6L. Primary, murine neurons were treated with the BACE inhibitor C3 or DMSO as a control. Proteins at the surface were labeled with biotin and enriched using streptavidin pull-down. Biotinylated SEZ6 and SEZ6L were detected by immunoblot. As a control, both proteins were also detected in whole cell lysates. Note, that only the mature 170 kDa form of SEZ6 was biotinylated at the cell surface. As a control, the ADAM10 substrate LDL receptor (LDLR) did not show a change in surface levels upon C3-treatment. As a further control, the cytosolic protein actin was only detected in whole lysates, but not among the surface biotinylated proteins

The deglycosylation experiment (Fig. 1b) had revealed that mature SEZ6 and SEZ6L carry complex N-linked sugars and are resistant to EndoH treatment. Complex sugars are added as proteins move through the Golgi apparatus. Thus, the mature forms of SEZ6 and SEZ6L are likely to be located in late compartments of the secretory pathway or at the plasma membrane. Indeed, using cell surface biotinylation the mature, but not the immature forms of both proteins were detected at the cell surface of primary neurons (Fig. 4). Treatment with the BACE inhibitor C3 increased full-length, mature SEZ6 and SEZ6L in whole cell lysates (Fig. 2b) and also at the cell surface (Fig. 4). As a control, surface levels of the LDL-receptor (LDLR), which is a substrate of ADAM10, but not of BACE1 [38], were not altered upon BACE inhibition. To demonstrate the specificity of the surface biotinylation, β-actin was detected in whole lysates, but strongly reduced in the pull-down of the biotinylated cell surface proteins (Fig. 4), as expected for a cytoplasmic protein. Taken together, BACE1 activity negatively controls the levels of SEZ6 and SEZ6L at the neuronal cell surface and in whole lysates.

### BACE1 cleaves SEZ6 within its juxtamembrane domain

**Fig. 5:**
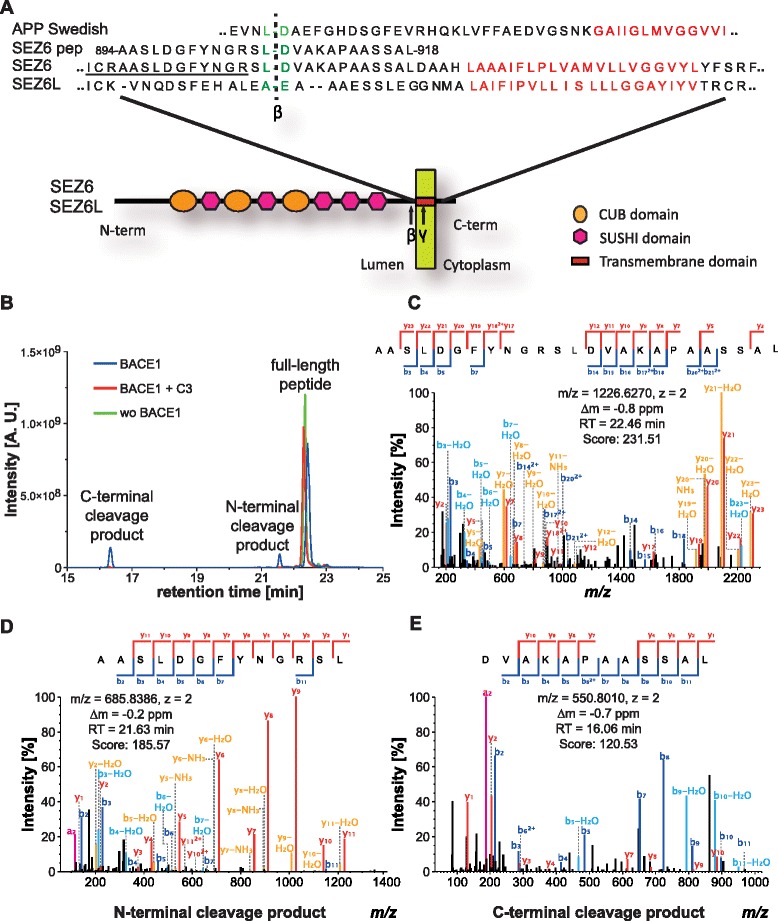
Cleavage site determination of SEZ6. **a** Comparison of BACE1 cleavage sites in the known APP Swedish mutant, in SEZ6 and SEZ6L. Additionally, the peptide (SEZ6 pep) used for the in vitro assay is aligned. Numbers next to the N- and C-terminal amino acids of the peptide indicate the amino acid number within the sequence of the full-length protein. Amino acids at the cleavage site are shown in *green*. Amino acids of the transmembrane domains are in *red*. Domains of SEZ6 and SEZ6L are shown with indicated symbols. The most C-terminal tryptic peptide of the secreted SEZ6 ectodomain detected in our previous study is underlined in *black*. **b** Extracted ion chromatogram of full-length peptide incubated with BACE1, BACE1 plus C3 or without BACE1 showing the peaks of the two cleavage products as well as the full-length peptide. Identification of the full-length peptide (**c**), the N-terminal (**d**) and the C-terminal cleavage product (**e**) by fragment ion spectra. The mapped y and b fragment ions are indicated in the sequences as well as in fragment ion spectra. Neutral loss fragment ions are indicated in *light blue* for b and *orange* for y ions

Next, we determined the cleavage site of BACE1 within the juxtamembrane domain of SEZ6 and compared it to the previously identified cleavage site within its homolog SEZ6L [15]. In the previous proteomic study which identified SEZ6 as a BACE1 substrate candidate, several tryptic peptides of the secreted SEZ6 ectodomain were identified. The most C-terminal of these peptides encompassed amino acids 894 to 904 (AASLDGFYNGR) of murine SEZ6 (Fig. 5a). This was a tryptic peptide ending with arginine (R), but BACE1 preferentially cleaves C-terminally to leucine or other hydrophobic amino acids [40]. Thus, the BACE1 cleavage site is likely to be located between this tryptic peptide and the transmembrane domain (start: leucine 923). To determine this site precisely, an in vitro peptide assay was used. The 25 amino acid peptide AASLDGFYNGRSLDVAKAPAASSAL (Fig. 5a, amino acids 894 to 918), comprising the tryptic peptide and ending shortly before the transmembrane domain, was incubated in the presence or absence of recombinant BACE1 with or without the BACE1 inhibitor C3 (Fig. 5b). Full-length peptide and cleavage fragments were separated by nano liquid chromatography and analyzed by high resolution mass spectrometry (nanoLC/MS). The non-cleaved, full-length peptide eluted from the nLC column at ~ 22 min (Fig. 5b). The correct sequence was verified by MS/MS-based fragmentation (Fig. 5c). Upon addition of BACE1, the full-length peptide levels were decreased in the chromatogram and two additional peptides with elution times of ~16 and ~21 min were detected (Fig. 5b). Addition of C3 inhibited the production of both peptides, demonstrating that they are BACE1 cleavage products of the full-length peptide. The two peptides were identified as AASLDGFYNGRSL (N-terminal cleavage product, Fig. 5d) and DVAKAPAASSAL (C-terminal cleavage product 2, Fig. 5e) by fragment spectra. Thus, we conclude that the BACE1 cleavage site in SEZ6 is the peptide bond between leucine906 and aspartate907 (Fig. 5a). Interestingly, this site comprises the same amino acids in the P1 and P1’ position (L-D) as Swedish mutant APP (Fig. 5a), which is very efficiently cleaved by BACE1 [4]. The previously identified cleavage site in SEZ6L [15] is not identical, but similar to SEZ6, as it also has a hydrophobic amino acid in the P1 and a negatively charged amino acid in the P1’ position (Fig. 5a). Moreover, SEZ6 and SEZ6L are both cleaved at a similar distance from the transmembrane domain, i.e. 16 and 14 amino acids for SEZ6 and SEZ6L, respectively (Fig. 5a).

### SEZ6 is a substrate for γ-secretase

**Fig. 6:**
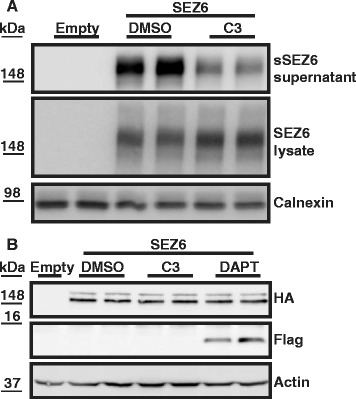
SEZ6 is a substrate for γ-secretase. **a** HEK293T cells were stably transfected with empty vector (Empty) or SEZ6 expression construct with an N-terminal HA-tag and a C-terminal FLAG epitope tag. Cells were treated with C3 or DMSO as a control. sSEZ6 was detected in the cell supernatant and full-length SEZ6 in the lysate. Calnexin was used as a loading control. **b** Cells were treated with DMSO, C3 or the γ-secretase inhibitor DAPT. The C-terminal SEZ6 fragment was detected by immunoblot using an anti-FLAG-tag antibody

After initial BACE1 cleavage, the resulting C-terminal, membrane-bound protein fragments of several membrane proteins, including APP and SEZ6L [17], are further processed within their transmembrane domains by γ-secretase, in a process referred to as regulated intramembrane proteolysis [41] (for schematic overview see Fig. 2a). The accumulation of C-terminal fragments upon pharmacological inhibition of γ-secretase with DAPT can be used to identify γ-secretase substrates [42]. To examine if SEZ6 is also cleaved by γ-secretase, we generated a human embryonic kidney 293 (HEK293T) cell line stably expressing murine SEZ6. Due to the lack of an antibody against the SEZ6 C-terminus, the full-length SEZ6 construct was tagged with an N-terminal HA and a C-terminal FLAG epitope tag. The full-length SEZ6 in the cell lysate and the shed ectodomain (sSEZ6) in the supernatant were detected by immunoblots in the transfected cells, but not in control transfected cells (Fig. 6a). Addition of the BACE inhibitor C3 decreased the sSEZ6 (Fig. 6a), in agreement with the results in neurons (Fig. 2b). The expected C-terminal fragment arising through BACE1 cleavage was not detected in control cells without the γ-secretase inhibitor DAPT, presumably because of its fast turnover. However, γ-secretase inhibition led to a strong accumulation of the SEZ6 C-terminal fragment at a molecular weight of around 13 kDa (Fig. 6b), which is consistent with the theoretical molecular weight of about 10 kDa for the C-terminal fragment starting at the BACE1 cleavage site and ending with the C-terminal FLAG-tag. These results indicate that SEZ6 is a γ-secretase substrate.

### sSEZ6 and sSEZ6L are detected in murine CSF in BACE1-dependent manner

Finally, we tested in vivo whether levels of sSEZ6 and sSEZ6L in murine CSF may be useful biomarkers for BACE1 activity in vivo. A previous proteomic study demonstrated that the soluble ectodomains of other BACE1 substrates, such as APLP1, PLXDC2 and CHL1, were reduced in the CSF of BACE1-deficient mice [14]. However, sSEZ6 and sSEZ6L were not consistently detected and could not be quantified in murine CSF, potentially because their levels were below the detection limit. Thus, we first improved the method for proteomic analysis of murine CSF in order to identify and quantify a larger number of proteins compared to the previous study. Most BACE1 inhibitors currently tested in clinical trials for AD are not specific for BACE1, but also inhibit BACE2. To mimic this situation we applied the improved proteomic method to the analysis of CSF from seven 4-month old BACE1/BACE2 double knock-out (BACE DKO) and seven age-matched WT mice.

In our previous protocol for mouse CSF proteomics, proteins were digested in the presence of urea and thiourea [14]. We replaced these nonionic chaotropes with the mild ionic detergent sodium deoxycholate (SDC), which has been shown to improve trypsin digestion of membrane proteins [43, 44]. A concentration of 0.1 % SDC was sufficient to improve the digestion efficiency. Triplicates of a pooled mouse CSF sample were digested with either the urea or the SDC-supported digestion protocol. The number of identified unique peptides was 6.6 % lower for the SDC supported protocol (Table 1). However, digestion efficiency was strongly increased which was detected by the 58.1 % lower number of average missed cleavages per peptide (Table 1). Additionally, the average number of identified and quantified proteins was 10.3 and 7.5 % higher for the SDC supported digestion protocol, respectively. Subcellular locations of proteins quantified in all replicates of SDC or urea supported digestions were similar (Additional file 1: Figures S2 and S3). However, the number of quantified membrane proteins was 8.9 % higher for the samples digested in the presence of SDC (135 vs. 124).

**Table 1:** Comparison of urea and SDC supported digestion of mouse CSF

	SDC	Urea	Difference
Unique peptides	5955.3	6376.7	−6.6 %
Average missed cleavages per peptide	0.26	0.62	−58.1 %
Protein identifications (≥2 unique peptides)	814.0	738.0	+10.3 %
Protein quantifications	847.7	788.3	+7.5 %

Values are averaged over three replicates

**Fig. 7:**
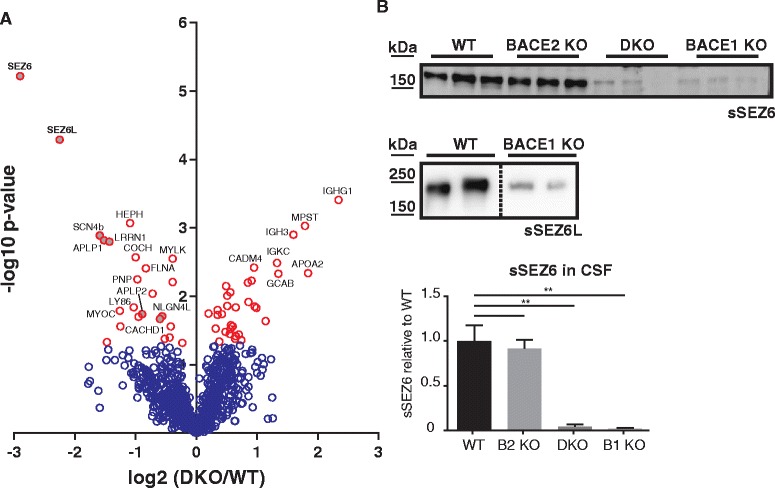
Proteomic analysis of CSF from BACE DKO and WT mice. **a** Volcano plot of proteomic analysis of BACE1 and BACE2 double knockout (BACE DKO) and WT mouse CSF. The minus log10 transformed t-test *p*-values are plotted against the log2 transformed label-free quantification intensity ratios of BACE DKO and WT CSF for every relatively quantified protein. Proteins with a t-test *p*-value < 0.05 are shown as *red circles*. Already known BACE substrate candidates with a *p*-value < 0.05 are marked with *gray* filling. Proteins that remain significant after Benjamini-Hochberg false discovery rate correction (FDR < 0.05) have bold letters (SEZ6 and SEZ6L). **b** Detection of sSEZ6 and sSEZ6L in mouse CSF. Densitometric quantitation of the Western blot is shown, (**; *p* < 0.01, one-way ANOVA followed by two-tailed Student’s t-Test, *n* = 3). The dotted line indicates that the samples were loaded onto the same blot, but not next to each other

Next, BACE DKO CSF was compared to WT CSF. In contrast to our previous proteomic study of CSF from BACE1 deficient mice [14], we were able to quantify SEZ6 and SEZ6L with the optimized protocol (Additional file 2: Supplementary Data: proteins BACE DKO vs WT CSF). The levels of several known or proposed BACE1 substrates such as SEZ6, SEZ6L, SCN4B, LRRN1, APLP1, APLP2, CACHD1 and NLGN4L were significantly reduced in BACE DKO CSF (Fig. 7a). Among these proteins, SEZ6 (DKO/WT = 13 %, *p* = 5.99E-06) and SEZ6L (DKO/WT = 20 %, *p* = 5.10E-05) showed the strongest reduction as well as the highest statistical significance (Fig. 7a). Changes in sSEZ6 and sSEZ6L also remained significant, when applying the Benjamini-Hochberg false discovery rate adjustment (α = 5 %) to correct for multiple hypothesis testing. In contrary, the third SEZ6 family member, SEZ6L2, did not show a significantly lower abundance in BACE DKO CSF, indicating that it is mostly cleaved by protease other than BACE1 or BACE2 (Fig. 7a).

Interestingly, the interleukin-6 receptor subunit beta (IL6ST) was quantified in all WT CSF samples by four unique peptides but in none of the BACE DKO CSF samples. This indicates that IL6ST may be an additional BACE1 and/or BACE2 substrate. Another BACE substrate candidate could be the type-1 transmembrane protein hephaestin (HEPH), which was significantly reduced by 53 % in BACE DKO CSF. Hephaestin is known to be expressed in the brain [45]. Additionally, peptide sequences of transmembrane and GPI-anchored proteins were loaded into the bioinformatics software tool QARIP [46] to check for their position within the protein sequences. Peptides were almost exclusively mapped to extracellular domains of transmembrane proteins (Additional file 3: Tables S1-S6). This indicates that most transmembrane proteins in the CSF are derived from proteolytic shedding and not from contaminating cells. For SEZ6, SEZ6L and SEZ6L2 only peptides from the ectodomain were identified (Additional file 3: Table S1).

To validate the proteomic results, reduced abundance of sSEZ6 and sSEZ6L were confirmed using immunoblots of independent CSF samples (Fig. 7b). In agreement with the proteomic analysis, sSEZ6 was nearly completely absent in BACE DKO CSF. The same reduction was observed in CSF from BACE1 KO, but not for BACE2 KO mice. This demonstrates that sSEZ6 is generated specifically by BACE1, but not by BACE2 in murine CSF. Likewise, sSEZ6L was strongly reduced in BACE1 KO CSF. Taken together, these results show that sSEZ6 and sSEZ6L levels can be used to monitor BACE1 activity in murine CSF.

## Discussion

BACE1 is a major drug target in AD, but has additional substrates and thus contributes to various biological processes [1, 13], which may limit its therapeutic potential. Recent proteomic studies have identified more than 40 membrane proteins as potential BACE1 substrates [14–16]. However, only few of them have been validated in vitro and in vivo. Using different techniques, our study validates SEZ6 and SEZ6L as BACE1 substrates in vitro and in vivo and demonstrates that, in contrast to other BACE1 substrates, SEZ6 and SEZ6L are nearly exclusively cleaved by BACE1 and not by other proteases in the brain. Levels of the soluble ectodomains (sSEZ6, sSEZ6L) were reduced to less than 10 % of the control levels upon pharmacological inhibition of BACE1 in primary neurons. Additionally, SEZ6 and SEZ6L were validated in vivo as BACE1 substrates using brains and CSF from BACE1 KO, BACE2 KO and/or BACE DKO mice. Thus, we propose that in addition to Aβ and sAPPβ, which are two BACE1 cleavage products of APP, sSEZ6 and sSEZ6L may be suitable as biomarkers to monitor BACE1 activity in vivo in CSF.

Several other previously identified BACE1 substrates, such as CHL1, L1, contactin-2, APP and its homolog APLP2, are not exclusively cleaved by BACE1, but also by other proteases, including ADAM10 [15, 38]. For example, the APP homolog APLP2 is cleaved to about 60 % by BACE1 and to 40 % by ADAM10 in neurons, but the percentages may strongly vary for each substrate [38]. Additionally, the different proteases may compensate for each other, if one of them is blocked. One example is APP. BACE1 inhibition increases the ADAM10 cleavage of APP, such that total APP cleavage is only mildly reduced [36, 47]. Potentially, this is also true for the third SEZ6 family member, SEZ6L2, which shows only moderately reduced shedding upon BACE1 inhibition [15]. A similar compensation does not occur for SEZ6 and SEZ6L in brain, where total cleavage was nearly completely abolished upon BACE1 inhibition. However, in other cell types and tissues both proteins may be cleaved by proteases different than BACE1. A previous study reported that in pancreatic cells SEZ6L is predominantly cleaved by BACE2, but not by BACE1 [17]. We confirm this finding and also extend it to SEZ6. Importantly, we show that neither SEZ6 nor SEZ6L are substrates for BACE1 in the pancreatic cell line, which is in contrast to brain, demonstrating that SEZ6 and SEZ6L are cleaved by different proteases in a tissue-specific manner. Precedents for such a tissue-specific proteolytic cleavage are the BACE1 substrates CHL1 and L1, which are mostly cleaved by BACE1 in the nervous system, but by ADAM proteases in non-neuronal cells [15, 37]. We found opposite expression patterns of BACE1 and BACE2 in MIN6 cells and in neurons, which correlated with the tissue-specific cleavage of SEZ6 and SEZ6L. Whether the distinct protease cleavage events also lead to a different functional outcome for the substrates remains to be investigated. This is particularly relevant as different proteases may cleave at distinct peptide bonds and thus generate ectodomains of different lengths and potentially different functions. For example, in APP the ADAM10 and BACE1 cleavage sites are 16 amino acids apart from each other and yield APP ectodomains with diverging functions [48, 49].

The molecular functions of SEZ6 and SEZ6L are not yet well understood. The name SEZ6 comes from the initial finding that SEZ6 expression was upregulated in cortical murine cells treated with the seizure-inducing drug pentylene tetrazole [32]. SEZ6 has been genetically linked to febrile seizures and epilepsy [50, 51], whereas SEZ6L was associated with bipolar disorder [52]. The extracellular regions of SEZ6/SEZ6L contain three CUB (complement subcomponent C1r, C1s /sea urchin embryonic growth factor Uegf / bone morphogenetic protein 1) and five short consensus repeat domains, which are protein-binding domains that are also found in a variety of cell surface receptors. This suggests that SEZ6/SEZ6L may act as receptors at the cell surface. Importantly, our study demonstrates that BACE1 cleavage negatively regulates SEZ6 and SEZ6L surface levels in neurons, suggesting that BACE1 may directly control SEZ6/SEZ6L surface functions. This could be a more general function of BACE1, because BACE1 also negatively regulates surface levels and/or function of two other substrates, contactin-2 and CHL1 [12, 15, 53]. However, the function of SEZ6 and SEZ6L may not only be exerted by the full-length proteins, but also by sSEZ6 and sSEZ6L or even by the C-terminal fragments resulting from BACE1 cleavage, as recently found for the BACE1 substrate CHL1 [12].

Future studies need to address how exactly BACE1 alters SEZ6 and SEZ6L function and whether such alterations contribute to the multiple phenotypes observed in BACE1-deficient mice. Notably, both BACE1- and SEZ6-deficient mice have deficits in hippocampal learning paradigms [18, 54–56] and in motor coordination [18, 57]. Moreover, both mouse lines appear to have reduced levels of anxiety and/or cognitive deficits [18, 56], reduced glutamatergic synapse function and reduced dendritic spine densities [18, 58]. Given the substantial overlap, at least some of these phenotypes may result from the reduced cleavage products of SEZ6/SEZ6L.

Another major outcome of our study is an improved protocol for efficient proteomic analysis of murine CSF. While human CSF is available in milliliter quantities, only approximately 10 μl of murine CSF are obtainable. Here, we improved the digestion efficiency of murine CSF in comparison to our previous protocol by using 0.1 % SDC in 50 mM ammonium bicarbonate as digestion buffer. This was demonstrated by the strong reduction of the average missed cleavages per peptide as well as the increased number of identified and quantified proteins (Table 1). The improved method may be of wide relevance for studying murine CSF in the context of different neurological and neurodegenerative diseases. Importantly, the new workflow allowed the quantification of SEZ6 and SEZ6L, which were not quantified in the previous study [14]. The nearly complete absence of sSEZ6 and sSEZ6L in murine CSF makes both cleavage products suitable markers to monitor BACE1 inhibition in mice. This may be particularly useful for determining the target engagement and potential side effects of BACE inhibitors in animal models. If confirmed in human CSF, sSEZ6 and sSEZ6L may even be useful as companion diagnostics to guide BACE inhibitor dosing in individual patients and monitor BACE1 inhibitor selectivity.

## Conclusions

We demonstrate that SEZ6 and SEZ6L are physiological BACE1 substrates in the murine brain and that, in contrast to most other BACE1 substrates, these two proteins are nearly exclusively cleaved by BACE1. Levels of sSEZ6 and sSEZ6L were strongly reduced upon pharmacological inhibition or genetic deficiency of BACE1 in primary neurons and mouse brain. Additionally, we developed an improved method for whole proteome analysis of murine CSF and found that in the CSF of BACE DKO mice the soluble ectodomains of SEZ6 and SEZ6L were most strongly reduced among all BACE1 substrates identified, suggesting their use as potential biomarkers in CSF to monitor BACE1 activity in vivo in mice.

## Additional files

Additional file 1: Figure S1. Comparison of BACE1 and BACE2 expression in MIN6 and WT primary neurons; **Figure S2**. UniProt subcellular location of proteins quantified in 3 out of 3 replicates of urea and SDC supported digestion; **Figure S3**. Sub-classification of membrane proteins quantified in three out of three replicates of urea and SDC supported digestion. (PDF 243 kb)
Additional file 2: Supplementary Data. Proteins and peptides identified and quantified in BACE DKO and WT CSF. This file contains four sheets, the first one of which contains the list and quantification of all proteins identified in the CSF. (XLSX 17716 kb)
Additional file 3: Tables S1-S6. They contain the proteins and peptides identified in BACE DKO and WT CSF. (PDF 918 kb)

## Abbreviations

AD: Alzheimer’s disease
APP: Amyloid precursor protein
Aβ: Amyloid β peptide
BACE1: β-site APP cleaving enzyme
BSA: Bovine Serum Albumin
CSF: Cerebrospinal fluid
DIV: Days in vitro
Endo H: Endoglycosidase H
HEK293T: Human embryonic kidney 293
HEPH: Hephaestin
IL6ST: Interleukin-6 receptor subunit beta
KO: Knock out
LDLR: LDL-receptor
LFQ: Label-free quantification
MSD: Mesoscale discovery
PBS: Phosphate buffered saline
PNGase F: Peptide-N-Glycosidase
SDC: Sodium deoxycholate
SEZ6: Seizure protein 6
SEZ6L: SEZ6-like
SEZ6L2: SEZ6-like 2
SLIC: Ligase independent cloning
sSEZ6, sSEZ6L: Soluble SEZ6 and SEZ6L
WT: Wild type
