# Supplementary material for: Seizure protein 6 and its homolog seizure 6-like protein are physiological substrates of BACE1 in neurons
Source: Mol Neurodegener. 2016 Oct 5;11:67. doi: 10.1186/s13024-016-0134-z (PMC5053352; doi:10.1186/s13024-016-0134-z)
Supplement: Additional file 1: Figure S1. — Comparison of BACE1 and BACE2 expression in MIN6 and WT primary neurons; Figure S2. UniProt subcellular location of proteins quantified in 3 out of 3 replicates of urea and SDC supported digestion; Figure S3. Sub-classification of membrane proteins quantified in three out of three replicates of urea and SDC supported digestion. (PDF 243 kb) [file 13024_2016_134_MOESM1_ESM.pdf]

Supplementary Information

Seizure protein 6 and its homolog seizure 6-like protein are physiological substrates of BACE1 in neurons

Martina Pigoni [1,2], Johanna Wanngren [1,2], Peer-Hendrik Kuhn [2,3,4], Kathryn M. Munro [5,] Jenny M. Gunnersen [5,6], Hiroshi Takeshima[7], Regina Feederle [8], Iryna Voytyuk [9], Bart De Strooper [9,10,11], Mikail D. Levasseur[12], Brian J. Hrupka[12], Stephan A. Müller [1,2] and Stefan F. Lichtenthaler [1,2,3,13]*

Affiliations

1: Deutsches Zentrum für Neurodegenerative Erkrankungen (DZNE), Munich, Germany
2: Neuroproteomics, Klinikum rechts der Isar, Technische Universität München, Munich, Germany;
3: Institute for Advanced Study, Technische Universität München, Munich, Germany;
4: Institut für Pathologie und Pathologische Anatomie, Technische Universität München, Munich, Germany
5: Department of Anatomy and Neuroscience, University of Melbourne, Victoria, Australia
6: The Florey Institute of Neuroscience and Mental Health, University of Melbourne, Victoria, Australia
7: Division of Pharmaceutical Sciences, Graduate School and Faculty of Pharmaceutical Sciences, Kyoto University, Japan
8: German Research Center for Environmental Health, Institute of Molecular Tumor immunology, Helmholtz Zentrum München, Munich, Germany.
9: VIB Center for the Biology of Disease, Leuven, Belgium
10: Center for Human Genetics, and Leuven Institute for Neurodegenerative Diseases (LIND), University of Leuven (KU Leuven), Leuven, Belgium
11: Institute of Neurology, University College London, London, UK
12: Department of Neuroscience, Janssen Pharmaceutica NV, Beerse, Belgium
13: Munich Cluster for Systems Neurology (SyNergy), Munich, Germany

*Correspondence should be sent to: stefan.lichtenthaler@dzne.de

Index

Suppl. Fig. 1: Comparison of BACE1 and BACE2 expression in MIN6 and WT primary neurons 3

Suppl. Fig. 2: UniProt subcellular location of proteins quantified in 3 out of 3 replicates of urea and SDC supported digestion 4

Suppl. Fig. 3: Sub-classification of membrane proteins quantified in 3 out of 3 replicates of urea and SDC supported digestion 5

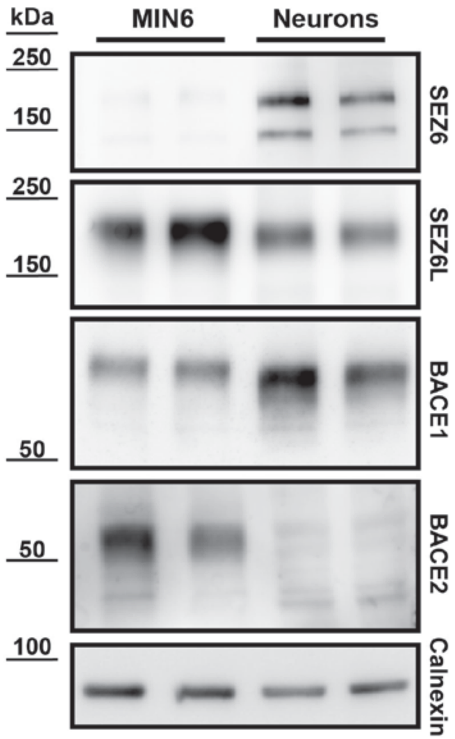

Suppl. Fig. 1: **Comparison of BACE1 and BACE2 expression in MIN6 and WT primary neurons.** Total lysates from MIN6 cells and primary WT neurons were probed with the indicated antibodies against SEZ6, SEZ6L, BACE1, BACE2 or calnexin. 15 µg of total lysate was used.

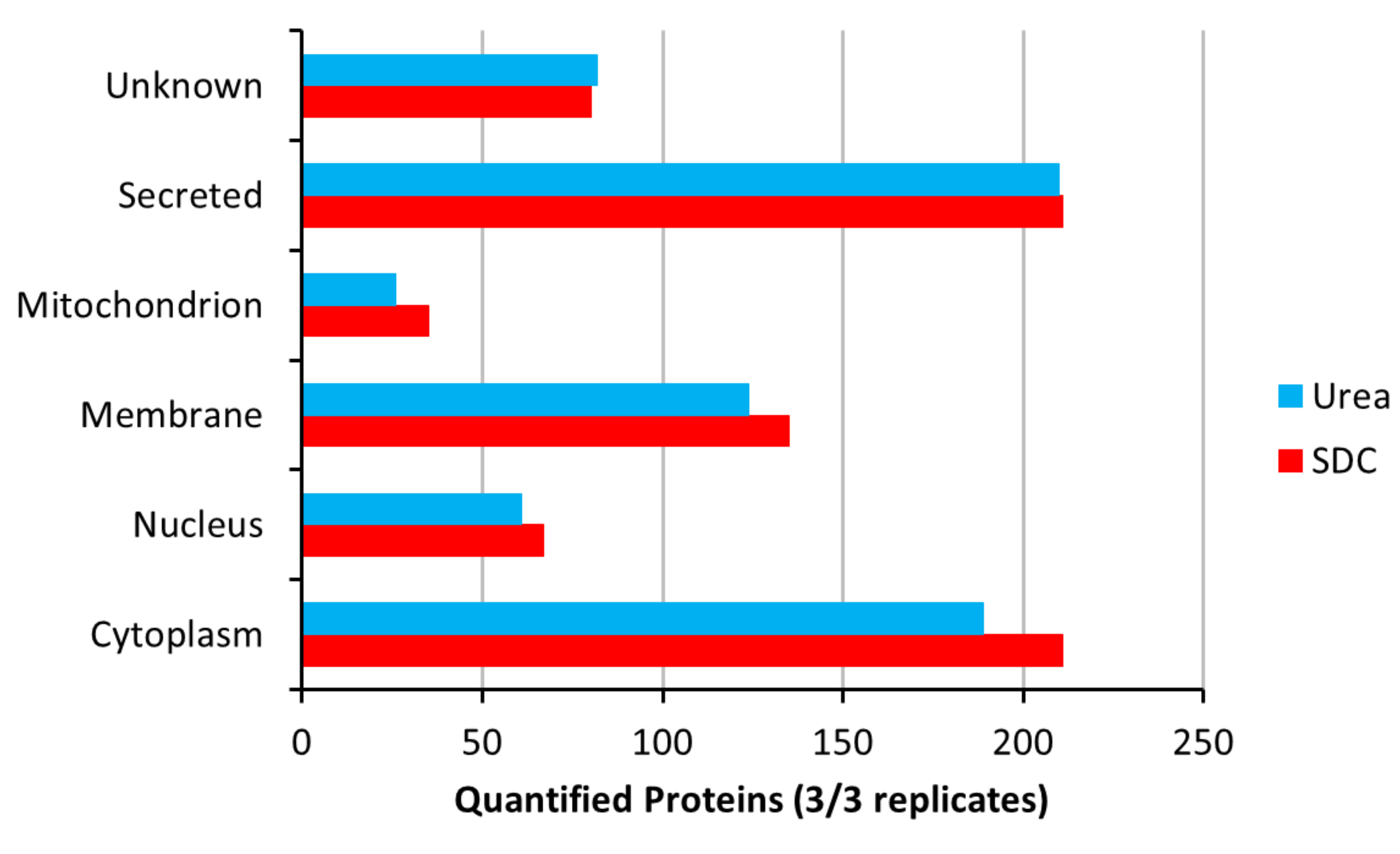

Suppl. Fig. 2: **UniProt subcellular location of proteins quantified in 3 out of 3 replicates of urea and SDC supported digestion.** (UniProt subcellular location terms: Cytoplasm [SL-0086], Nucleus [SL-0191], Membrane [SL-0162], Mitochondrion [SL-0173], Secreted [SL-0243])

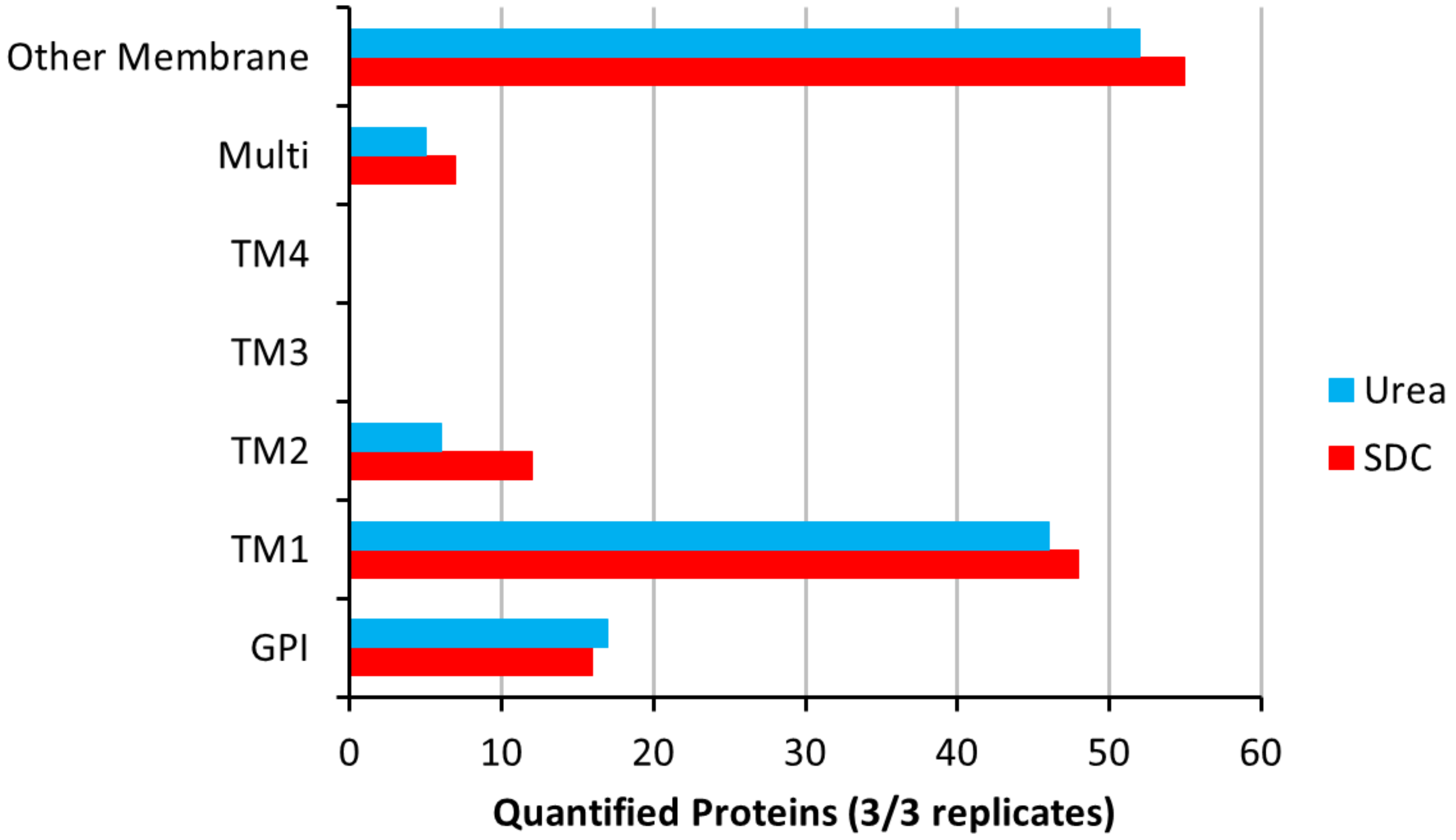

Suppl. Fig. 3: **Sub-classification of membrane proteins quantified in 3 out of 3 replicates of urea and SDC supported digestion.** Following UniProt subclasses were used: GPI-anchored proteins (GPI), single-pass membrane type 1 (TM1), type2 (TM2), type 3 (TM3), type 4 (TM4) and multi-pass membrane proteins (Multi). Membrane proteins that were not sub-classified in one of the groups are indicated as “Other Membrane”. (UniProt subcellular location terms: GPI-anchor [SL-9902], Single-pass type I membrane protein [SL-9905], Single-pass type II membrane protein [SL-9906], Single-pass type III membrane protein [SL-9907], Single-pass type IV membrane protein [SL-9908], Multi-pass membrane protein [SL-9909])
