# Supplementary material for: Seizure protein 6 and its homolog seizure 6-like protein are physiological substrates of BACE1 in neurons
Source: Mol Neurodegener. 2016 Oct 5;11:67. doi: 10.1186/s13024-016-0134-z (PMC5053352; doi:10.1186/s13024-016-0134-z)
Supplement: Additional file 3: Tables S1-S6. — They contain the proteins and peptides identified in BACE DKO and WT CSF. (PDF 918 kb) [file 13024_2016_134_MOESM3_ESM.pdf]

Supplementary Information

Seizure protein 6 and its homolog seizure 6-like protein are physiological substrates of BACE1 in neurons

Martina Pigoni [1,2], Johanna Wanngren [1,2], Peer-Hendrik Kuhn [2,3,4], Kathryn M. Munro [5,] Jenny M. Gunnersen [5,6], Hiroshi Takeshima[7], Regina Feederle [8], Iryna Voytyuk [9], Bart De Strooper [9,10,11], Mikail D. Levasseur[12], Brian J. Hrupka[12], Stephan A. Müller [1,2] and Stefan F. Lichtenthaler [1,2,3,13]*

Affiliations

1: Deutsches Zentrum für Neurodegenerative Erkrankungen (DZNE), Munich, Germany
2: Neuroproteomics, Klinikum rechts der Isar, Technische Universität München, Munich, Germany;
3: Institute for Advanced Study, Technische Universität München, Munich, Germany;
4: Institut für Pathologie und Pathologische Anatomie, Technische Universität München, Munich, Germany
5: Department of Anatomy and Neuroscience, University of Melbourne, Victoria, Australia
6: The Florey Institute of Neuroscience and Mental Health, University of Melbourne, Victoria, Australia
7: Division of Pharmaceutical Sciences, Graduate School and Faculty of Pharmaceutical Sciences, Kyoto University, Japan
8: German Research Center for Environmental Health, Institute of Molecular Tumor immunology, Helmholtz Zentrum München, Munich, Germany.
9: VIB Center for the Biology of Disease, Leuven, Belgium
10: Center for Human Genetics, and Leuven Institute for Neurodegenerative Diseases (LIND), University of Leuven (KU Leuven), Leuven, Belgium
11: Institute of Neurology, University College London, London, UK
12: Department of Neuroscience, Janssen Pharmaceutica NV, Beerse, Belgium
13: Munich Cluster for Systems Neurology (SyNergy), Munich, Germany

*Correspondence should be sent to: stefan.lichtenthaler@dzne.de

Index

Suppl. Tab. 1: Identified transmembrane type 1 proteins in BACE DKO and WT CSF ____ 3-10

Suppl. Tab. 2: Identified transmembrane type 2 proteins in BACE DKO and WT CSF ____ 3-10

Suppl. Tab. 3: Identified transmembrane type 3 proteins in BACE DKO and WT CSF ____ 11

Suppl. Tab. 4: Identified transmembrane type 4 proteins in BACE DKO and WT CSF ____ 11

Suppl. Tab. 5: Identified multi-pass transmembrane proteins in BACE DKO and WT CSF ____ 12-13

Suppl. Tab. 6: Identified GPI-anchored proteins in BACE DKO and WT CSF ____ 14-15

Color code for schematic sequence in in the column Topology (derived by QARIP http://webclu.bio.wzw.tum.de/qarip/):

Signalpeptide

Extracellular domain

Transmembrane domain

Cytoplasmic domain

Lumenaldomain

Propeptide

Unknown

detected peptides

Suppl. Tab. 2: **Identified transmembrane type 1 proteins in BACE DKO and WT CSF.** (UniProt subcellular location: Single-pass type I membrane protein [SL-9905])

UniProt AC	Protein names	Gene names	Unique peptides	Ratio (DKO/WT)	p-value	Topology (UniProt)
Q7TSK2	Seizure protein 6	Sez6	13	0.13	5.99E-06	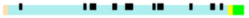
Q6P1D5	Seizure 6-like protein	Sez6l	6	0.21	5.10E-05	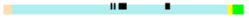
Q7M729	Sodium channel subunit beta-4	Scn4b	3	0.33	1.29E-03	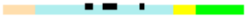
Q03157	Amyloid-like protein 1;C30	Aplp1	28	0.35	1.51E-03	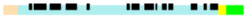
O54901	OX-2 membrane glycoprotein	Cd200	4	0.36	NaN	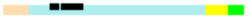
Q61809	Leucine-rich repeat neuronal protein 1	Lrrn1	7	0.37	1.58E-03	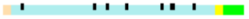
Q8BHC0	Lymphatic vessel endothelial hyaluronic acid receptor 1	Lyve1	3	0.43	NaN	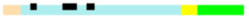
Q9Z0Z4	Hephaestin	Heph	4	0.47	8.51E-04	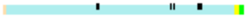
Q06335	Amyloid-like protein 2	Aplp2	17	0.54	1.83E-02	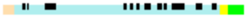
O35988	Syndecan-4	Sdc4	2	0.63	NaN	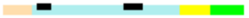
P16092	Fibroblast growth factor receptor 1	Fgfr1	7	0.63	6.81E-02	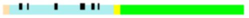
Q6PDJ1	VWFA and cache domain-containing protein 1	Cachd1	12	0.66	2.16E-02	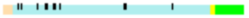

UniProt AC	Protein names	Gene names	Unique peptides	Ratio (DKO/WT)	p-value	Topology (UniProt)
B0F2B4	Neuroligin 4-like	Nlgn4l	9	0.68	1.96E-02	
Q61790	Lymphocyte activation gene 3 protein	Lag3	9	0.72	7.88E-02	
Q9DC11	Plexin domain-containing protein 2	Plxdc2	8	0.74	1.94E-01	
E9Q7X6	Protein HEG homolog 1	Heg1	11	0.74	3.38E-01	
Q61830	Macrophage mannose receptor 1	Mrc1	5	0.75	2.74E-02	
Q8BI84	Melanoma inhibitory activity protein 3	Mia3	2	0.75	4.36E-01	
Q61490	CD166 antigen	Alcam	3	0.77	6.21E-03	
P42703	Leukemia inhibitory factor receptor	Lifr	18	0.78	3.88E-01	
P11627	Neural cell adhesion molecule L1	L1cam	14	0.79	2.85E-01	
Q61851	Fibroblast growth factor receptor 3	Fgfr3	6	0.81	1.73E-01	
P70232	Neural neural cell adhesion molecule L1-like protein	Chl1	33	0.81	2.96E-01	
Q64449	C-type mannose receptor 2	Mrc2	14	0.81	9.18E-02	
Q9D7I0	Protein shisa-5	Shisa5	2	0.84	NaN	
Q4V9Z5	Seizure 6-like protein 2	Sez6l2	14	0.85	5.03E-01	
Q9R1V6	Disintegrin and metalloproteinase domain-containing protein 22	Adam22	6	0.87	4.84E-01	
Q99N28	Cell adhesion molecule 3	Cadm3	8	0.88	5.42E-01	
O08644	Ephrin type-B receptor 6	Ephb6	8	0.88	3.80E-01	
P09803	Cadherin-1;E-Cad/CTF1;E-Cad/CTF2;E-Cad/CTF3	Cdh1	3	0.89	5.27E-01	
P12023	Amyloid beta A4 protein	App	31	0.89	6.54E-01	
Q6AZB0	Brother of CDO	Boc	1	0.90	3.36E-01	
Q9ET66	Peptidase inhibitor 16	Pi16	5	0.92	4.66E-01	
P09470	Angiotensin-converting enzyme	Ace	32	0.92	6.36E-01	
O88307	Sortilin-related receptor	Sorl1	2	0.93	7.13E-01	
Q810U3	Neurofascin	Nfasc	16	0.94	6.89E-01	
O35393	Ephrin-B3	Efnb3	10	0.96	8.53E-01	
Q09163	Protein delta homolog 1;Fetal antigen 1	Dlk1	2	0.96	NaN	
O35516	Neurogenic locus notch homolog protein 2	Notch2	2	0.96	NaN	
Q91V98	Endosialin	Cd248	3	0.97	6.71E-01	
Q7TMJ8	Phosphoinositide-3-kinase-interacting protein 1	Pik3ip1	7	0.97	8.91E-01	
Q9JKF6	Nectin-1	Pvrl1	3	0.97	NaN	
Q03137	Ephrin type-A receptor 4	Epha4	14	0.98	9.20E-01	
Q8VHY0	Chondroitin sulfate proteoglycan 4	Cspg4	4	0.98	8.29E-01	
Q6PHS9	Voltage-dependent calcium channel subunit alpha-2/delta-2	Cacna2d2	12	0.98	9.29E-01	

UniProt AC	Protein names	Gene names	Unique peptides	Ratio (DKO/WT)	p-value	Topology (UniProt)
Q8VI51	VPS10 domain-containing receptor SorCS3	Sorcs3	7	0.99	9.40E-01	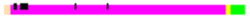
Q62313; Q62314	Trans-Golgi network integral membrane protein 1;Trans-Golgi network integral membrane protein 2	Tgoln1; Tgoln2	2	0.99	NaN	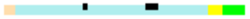
P17047	Lysosome-associated membrane glycoprotein 2	Lamp2	3	1.00	NaN	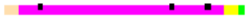
P09581	Macrophage colony-stimulating factor 1 receptor	Csf1r	9	1.01	9.73E-01	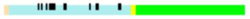
P80560	Receptor-type tyrosine-protein phosphatase N2	Ptprn2	17	1.01	9.77E-01	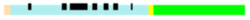
Q01279	Epidermal growth factor receptor	Egfr	16	1.01	9.83E-01	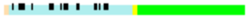
Q64487	Receptor-type tyrosine-protein phosphatase delta	Ptprd	4	1.02	8.63E-01	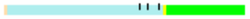
Q62179	Semaphorin-4B	Sema4b	9	1.03	9.17E-01	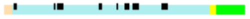
P13595	Neural cell adhesion molecule 1	Ncam1	24	1.03	8.81E-01	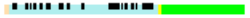
Q9R1V7	Disintegrin and metalloproteinase domain-containing protein 23	Adam23	5	1.05	7.17E-01	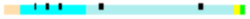
Q9EPR5	VPS10 domain-containing receptor SorCS2	Sorcs2	2	1.05	8.47E-01	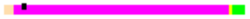
Q922P8	Transmembrane protein 132A	Tmem132a	23	1.05	8.72E-01	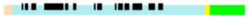
P18572	Basigin	Bsg	2	1.05	NaN	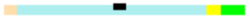
Q9CPW0	Contactin-associated protein-like 2	Cntnap2	6	1.06	6.82E-01	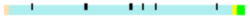
O35188	Fractalkine;Processed fractalkine	Cx3cl1	3	1.07	6.70E-01	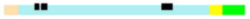
Q8BX43	Tumor necrosis factor receptor superfamily member 19L	Relt	2	1.08	7.57E-01	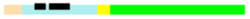
Q8CJH3	Plexin-B1	Plxnb1	3	1.08	5.24E-01	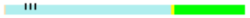
P55288	Cadherin-11	Cdh11	8	1.09	5.01E-01	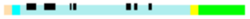
P15116	Cadherin-2	Cdh2	15	1.09	7.26E-01	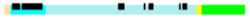
Q9ER65	Calsyntenin-2	Clstn2	3	1.09	NaN	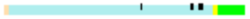
Q91ZX7	Prolow-density lipoprotein receptor-related protein 1	Lrp1	41	1.09	5.67E-01	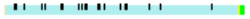
P11438	Lysosome-associated membrane glycoprotein 1	Lamp1	5	1.09	5.73E-01	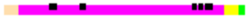
P98156	Very low-density lipoprotein receptor	Vldlr	3	1.10	6.18E-01	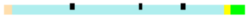
Q9JLC4	VPS10 domain-containing receptor SorCS1	Sorcs1	12	1.10	6.35E-01	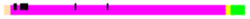
Q8R5M8	Cell adhesion molecule 1	Cadm1	8	1.11	6.17E-01	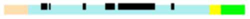
O35664	Interferon alpha/beta receptor 2	Ifnar2	4	1.11	5.23E-01	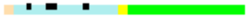
P29533	Vascular cell adhesion protein 1	Vcam1	13	1.12	5.22E-01	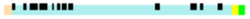
O88393	Transforming growth factor beta receptor type 3	Tgfbr3	10	1.12	6.08E-01	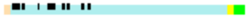
Q9CZT5	Vasorin	Vasn	7	1.13	5.30E-01	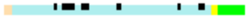
Q9Z1L5	Voltage-dependent calcium channel subunit alpha-2/delta-3	Cacna2d3	20	1.13	5.95E-01	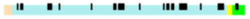
P97333	Neuropilin-1	Nrp1	6	1.13	1.09E-01	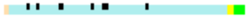
Q99JH7	Calsyntenin-3	Clstn3	16	1.14	5.62E-01	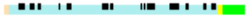

UniProt AC	Protein names	Gene names	Unique peptides	Ratio (DKO/WT)	p-value	Topology (UniProt)
Q6P1B3	PILR alpha-associated neural protein	Pianp	2	1.15	3.07E-01	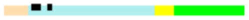
P15379	CD44 antigen	Cd44	2	1.15	1.76E-02	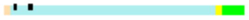
Q6P9K9	Neurexin-3	Nrxn3	26	1.16	5.02E-01	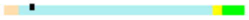
Q62165	Dystroglycan;Alpha-dystroglycan;Beta-dystroglycan	Dag1	13	1.17	5.34E-01	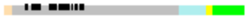
O89026	Roundabout homolog 1	Robo1	7	1.17	1.28E-01	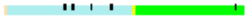
Q60673	Receptor-type tyrosine-protein phosphatase-like N	Ptprn	8	1.18	5.47E-01	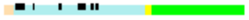
P39038	Cadherin-4	Cdh4	7	1.18	5.13E-01	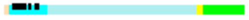
Q9EPL2	Calsyntenin-1;Soluble Alc-alpha;CTF1-alpha	Clstn1	34	1.18	4.38E-01	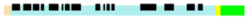
B2RXS4	Plexin-B2	Plxnb2	10	1.18	3.53E-01	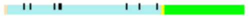
Q810B7	SLIT and NTRK-like protein 5	Slitrk5	3	1.18	3.00E-01	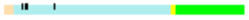
Q8BGX3	Leucine-rich repeat and transmembrane domain-containing protein 2	Lrtm2	5	1.18	2.05E-01	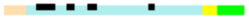
Q9CYN9	Renin receptor	Atp6ap2	11	1.19	5.91E-01	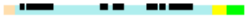
P05532	Mast/stem cell growth factor receptor Kit	Kit	4	1.19	NaN	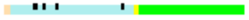
B0V2N1	Receptor-type tyrosine-protein phosphatase S	Ptprs	4	1.19	4.37E-01	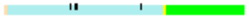
Q810C1	SLIT and NTRK-like protein 1	Slitrk1	7	1.19	4.49E-01	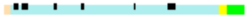
Q9EPU5	Tumor necrosis factor receptor superfamily member 21	Tnfrsf21	9	1.19	4.38E-01	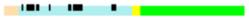
P35951	Low-density lipoprotein receptor	Ldlr	8	1.20	2.33E-01	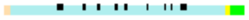
O35136	Neural cell adhesion molecule 2	Ncam2	5	1.21	3.95E-01	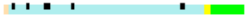
O08532	Voltage-dependent calcium channel subunit alpha-2/delta-1	Cacna2d1	33	1.21	4.12E-01	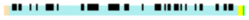
Q7TSK3	Protocadherin-8	Pcdh8	6	1.22	1.34E-01	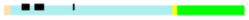
Q8BYM5	Neuroligin-3	Nlgn3	4	1.23	1.05E-01	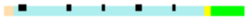
Q8BH27	Multiple epidermal growth factor-like domains protein 9	Megf9	2	1.23	2.07E-01	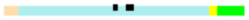
P33146	Cadherin-15	Cdh15	7	1.23	3.78E-01	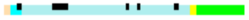
Q6IEE6	Transmembrane protein 132E	Tmem132e	9	1.24	2.11E-01	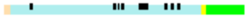
P07141	Macrophage colony-stimulating factor 1	Csf1	6	1.24	2.59E-01	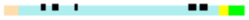
Q9CR75	Tumor necrosis factor receptor superfamily member 12A	Tnfrsf12a	3	1.24	1.13E-01	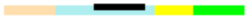
P35822	Receptor-type tyrosine-protein phosphatase kappa	Ptprk	5	1.25	2.96E-02	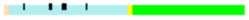
P97300	Neuroplastin	Nptn	5	1.25	1.85E-01	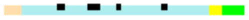
Q9D1T0	Leucine-rich repeat and immunoglobulin-like domain-containing nogo receptor-interacting protein 1	Lingo1	10	1.25	3.51E-01	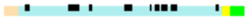
Q61730	Interleukin-1 receptor accessory protein	Il1rap	19	1.26	4.15E-01	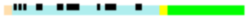
Q8CEF9	Transmembrane protein 132C	Tmem132c	4	1.27	1.01E-01	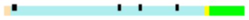
B9EKR1	Receptor-type tyrosine-protein phosphatase zeta	Ptprz1	22	1.28	3.03E-01	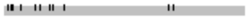

UniProt AC	Protein names	Gene names	Unique peptides	Ratio (DKO/WT)	p-value	Topology (UniProt)
Q810B8	SLIT and NTRK-like protein 4	Slitrk4	6	1.29	2.67E-01	
Q05909	Receptor-type tyrosine-protein phosphatase gamma	Ptprg	3	1.30	4.62E-02	
Q9CS84	Neurexin-1	Nrxn1	26	1.32	1.28E-01	
Q810C0	SLIT and NTRK-like protein 2	Slitrk2	2	1.32	NaN	
Q76KF0	Semaphorin-6D	Sema6d	13	1.34	2.13E-01	
Q9JME9	V-set and transmembrane domain-containing protein 2B	Vstm2b	4	1.36	7.11E-02	
Q6DIB5	Multiple epidermal growth factor-like domains protein 10	Megf10	14	1.39	5.68E-02	
P52800	Ephrin-B2	Efnb2	2	1.40	3.32E-02	
P97291	Cadherin-8	Cdh8	6	1.40	7.09E-03	
Q8JZM4	Delta and Notch-like epidermal growth factor-related receptor	Dner	6	1.40	8.97E-02	
Q8BT18	Uncharacterized protein C14orf37 homolog		3	1.42	1.09E-01	
Q9R1V4	Disintegrin and metalloproteinase domain-containing protein 11	Adam11	3	1.45	NaN	
Q810U4	Neuronal cell adhesion molecule	Nrcam	22	1.46	1.52E-01	
Q99K10	Neuroligin-1	Nlgn1	2	1.47	7.25E-02	
O35930	Platelet glycoprotein Ib alpha chain	Gp1ba	7	1.47	NaN	
Q99P47	Contactin-associated protein-like 4	Cntnap4	22	1.50	1.95E-01	
Q80TG9	Leucine-rich repeat and fibronectin type-III domain-containing protein 2	Lrfn2	3	1.54	NaN	
P97798	Neogenin	Neo1	6	1.57	1.45E-02	
O54951	Semaphorin-6B	Sema6b	5	1.59	9.75E-02	
P35546	Proto-oncogene tyrosine-protein kinase receptor Ret	Ret	3	1.64	NaN	
P01898	H-2 class I histocompatibility antigen, Q10 alpha chain	H2-Q10	7	1.74	1.80E-01	
Q810B9	SLIT and NTRK-like protein 3	Slitrk3	4	1.88	5.96E-03	
Q8R464	Cell adhesion molecule 4	Cadm4	4	1.93	3.82E-03	
Q9DBH5	Vesicular integral-membrane protein VIP36	Lman2	3	2.88	NaN	
Q00560	Interleukin-6 receptor subunit beta	Il6st	4	NaN	NaN	
P10404	MLV-related proviral Env polyprotein		6	NaN	NaN	
Q99NH8	Triggering receptor expressed on myeloid cells 2	Trem2	2	NaN	NaN	
Q9JHJ8	ICOS ligand	Icoslg	3	NaN	NaN	
O89103	Complement component C1q receptor	Cd93	3	NaN	NaN	
P09055	Integrin beta-1	Itgb1	3	NaN	NaN	
O35464	Semaphorin-6A	Sema6a	2	NaN	NaN	
O35598	Disintegrin and metalloproteinase domain-containing protein 10	Adam10	1	NaN	NaN	
O88324	CD83 antigen	Cd83	1	NaN	NaN	

UniProt AC	Protein names	Gene names	Unique peptides	Ratio (DKO/WT)	p-value	Topology (UniProt)
P14430	H-2 class I histocompatibility antigen, Q8 alpha chain	H2-Q8	1	NaN	NaN	
P15209	BDNF/NT-3 growth factors receptor	Ntrk2	1	NaN	NaN	
P20917	Myelin-associated glycoprotein	Mag	2	NaN	NaN	
P25118	Tumor necrosis factor receptor superfamily member 1A	Tnfrsf1a	1	NaN	NaN	
P26618	Platelet-derived growth factor receptor alpha	Pdgfra	2	NaN	NaN	
P28825	Meprin A subunit alpha	Mep1a	1	NaN	NaN	
P28828	Receptor-type tyrosine-protein phosphatase mu	Ptprm	1	NaN	NaN	
P55284	Cadherin-5	Cdh5	8	NaN	NaN	
P70408	Cadherin-10	Cdh10	3	NaN	NaN	
P97326	Cadherin-6	Cdh6	3	NaN	NaN	
P97797	Tyrosine-protein phosphatase non-receptor type substrate 1	Sirpa	1	NaN	NaN	
P97952	Sodium channel subunit beta-1	Scn1b	1	NaN	NaN	
Q03146	Epithelial discoidin domain-containing receptor 1	Ddr1	2	NaN	NaN	
Q06186	Proheparin-binding EGF-like growth factor	Hbegf	1	NaN	NaN	
Q3UH99	Protein shisa-6 homolog	Shisa6	1	NaN	NaN	
Q5SZV5	Dyslexia-associated protein KIAA0319 homolog	Kiaa0319	1	NaN	NaN	
Q60805	Tyrosine-protein kinase Mer	Mertk	1	NaN	NaN	
Q61772	Ephrin type-A receptor 7	Epha7	4	NaN	NaN	
Q64314	Hematopoietic progenitor cell antigen CD34	Cd34	2	NaN	NaN	
Q69ZN6	N-acetylglucosamine-1-phosphotransferase subunits alpha/beta	Gnptab	1	NaN	NaN	
Q6PHU5	Sortilin	Sort1	2	NaN	NaN	
Q71M36	Chondroitin sulfate proteoglycan 5	Cspg5	2	NaN	NaN	
Q8BIF0	CD99 antigen-like protein 2	Cd99l2	1	NaN	NaN	
Q8BLQ9	Cell adhesion molecule 2	Cadm2	4	NaN	NaN	
Q8BSU2	C-X-C motif chemokine 16	Cxcl16	1	NaN	NaN	
Q8C985	Neurexin-3-beta;Neurexin-3-beta	Nrxn3	1	NaN	NaN	
Q8CAE9	Podocalyxin-like protein 2	Podxl2	1	NaN	NaN	
Q8CB67	Low-density lipoprotein receptor-related protein 11	Lrp11	2	NaN	NaN	
Q8R2Y2	Cell surface glycoprotein MUC18	Mcam	1	NaN	NaN	
Q9D0F3	Protein ERGIC-53	Lman1	3	NaN	NaN	
Q9QZ26	Potassium voltage-gated channel subfamily E regulatory beta subunit 5	Kcne5	1	NaN	NaN	
Q9WU03	Kunitz-type protease inhibitor 2	Spint2	1	NaN	NaN	

Suppl. Tab. 2: **Identified transmembrane type 2 proteins in BACE DKO and WT CSF** (UniProt subcellular location: Single-pass type II membrane protein [SL-9906])

UniProt AC	Protein names	Gene names	Unique peptides	Ratio (DKO/WT)	p-value	Topology (UniProt)
F6VAN0	Cyclic AMP-dependent transcription factor ATF-6 alpha;Processed cyclic AMP-dependent transcription factor ATF-6 alpha	Atf6	2	0.51	1.38E-01	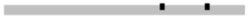
P14094	Sodium/potassium-transporting ATPase subunit beta-1	Atp1b1	1	0.57	NaN	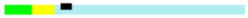
Q6P1J0	Glycoprotein endo-alpha-1,2-mannosidase-like protein	Maneal	3	0.68	NaN	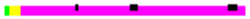
P97370	Sodium/potassium-transporting ATPase subunit beta-3	Atp1b3	3	0.70	5.09E-01	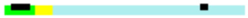
P10852	4F2 cell-surface antigen heavy chain	Slc3a2	5	0.86	4.74E-02	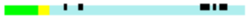
Q3U4G3	Xyloside xylosyltransferase 1	Xxylt1	11	0.92	5.84E-01	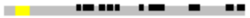
Q8BQ86	Carbohydrate sulfotransferase 8	Chst8	2	0.93	6.63E-01	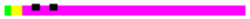
Q922Q6	Alpha/beta hydrolase domain-containing protein 14A	Abhd14a	3	0.97	7.06E-01	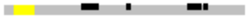
Q10470	Beta-1,4-mannosyl-glycoprotein 4-beta-N-acetylglucosaminyltransferase	Mgat3	2	1.00	NaN	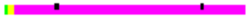
Q91XQ5	Carbohydrate sulfotransferase 15	Chst15	7	1.00	9.82E-01	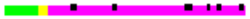
Q8CF93	Polypeptide N-acetylgalactosaminyltransferase 13	Galnt13	3	1.01	9.45E-01	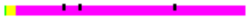
O89051	Integral membrane protein 2B;BRI2, membrane form;BRI2 intracellular domain;BRI2C, soluble form;Bri23 peptide	Itm2b	8	1.01	9.60E-01	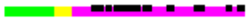
Q921V5	Alpha-1,6-mannosyl-glycoprotein 2-beta-N-acetylglucosaminyltransferase	Mgat2	6	1.03	8.65E-01	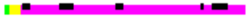
P15535	Beta-1,4-galactosyltransferase 1;Lactose synthase A protein	B4galt1	5	1.03	8.14E-01	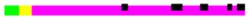
Q8BWP8	Beta-1,4-glucuronyltransferase 1	B3gnt1	12	1.06	8.23E-01	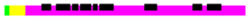
A2ASQ1	Agrin	Agrn	9	1.08	7.35E-01	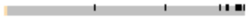
Q8VCF1	Soluble calcium-activated nucleotidase 1	Cant1	13	1.08	6.41E-01	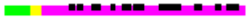
O09010	Beta-1,3-N-acetylglucosaminyltransferase lunatic fringe	Lfng	16	1.08	7.43E-01	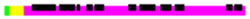
Q811B1	Xylosyltransferase 1	Xylt1	3	1.09	5.43E-01	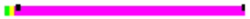
O35405	Phospholipase D3	Pld3	6	1.10	6.40E-01	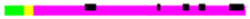
P45700	Mannosyl-oligosaccharide 1,2-alpha-mannosidase IA	Man1a1	19	1.11	6.33E-01	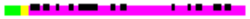
Q9EQH2	Endoplasmic reticulum aminopeptidase 1	Erap1	6	1.14	8.28E-02	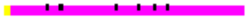
Q80V26	Inositol monophosphatase 3	Impad1	5	1.18	5.45E-01	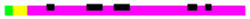
Q99J85	Neuronal pentraxin receptor	Nptxr	17	1.19	3.98E-01	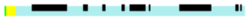
Q9JJ61	Polypeptide N-acetylgalactosaminyltransferase 16	Galntl1	3	1.21	3.02E-01	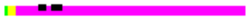
Q6PB93	Polypeptide N-acetylgalactosaminyltransferase 2	Galnt2	3	1.21	2.11E-01	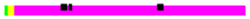
Q8R4G6	Alpha-1,6-mannosylglycoprotein 6-beta-N-acetylglucosaminyltransferase A	Mgat5	11	1.22	5.02E-01	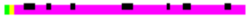
Q8K4Q8	Collectin-12	Colec12	3	1.23	2.27E-01	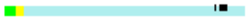
O08912	Polypeptide N-acetylgalactosaminyltransferase 1	Galnt1	8	1.24	1.02E-01	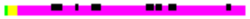
O88587	Catechol O-methyltransferase	Comt	4	1.25	NaN	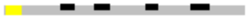

UniProt AC	Protein names	Gene names	Unique peptides	Ratio (DKO/WT)	p-value	Topology (UniProt)
Q09200	Beta-1,4 N-acetylgalactosaminyltransferase 1	B4galnt1	3	1.26	NaN	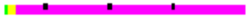
Q62351	Transferrin receptor protein 1	Tfrc	14	1.27	7.12E-02	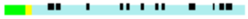
Q91X88	Protein O-linked-mannose beta-1,2-N-acetylglucosaminyltransferase 1	Pomgnt1	8	1.30	8.66E-02	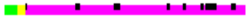
Q3UHN9	Bifunctional heparan sulfate N-deacetylase/N-sulfotransferase 1	Ndst1	7	1.30	2.35E-01	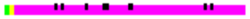
P70428	Exostosin-2	Ext2	5	1.43	5.96E-02	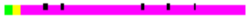
P70126	Alpha-2,8-sialyltransferase 8E	St8sia5	4	1.46	5.94E-02	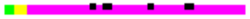
Q8BXA1	Golgi integral membrane protein 4	Golim4	2	1.46	3.54E-02	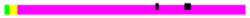
Q9ES89	Exostosin-like 2	Extl2	9	1.55	8.23E-02	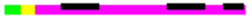
Q8BRK9	Alpha-mannosidase 2x	Man2a2	17	1.62	3.66E-02	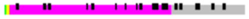
Q8VIB3	Type 2 lactosamine alpha-2,3-sialyltransferase	St3gal6	2	NaN	NaN	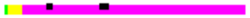
Q62092	Neuron-specific protein family member 1	Nsg1	1	NaN	NaN	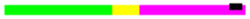
Q91VK4	Integral membrane protein 2C;CT-BRI3	Itm2c	1	NaN	NaN	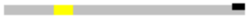
P39098	Mannosyl-oligosaccharide 1,2-alpha-mannosidase IB	Man1a2	1	NaN	NaN	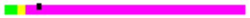
P27046	Alpha-mannosidase 2	Man2a1	5	NaN	NaN	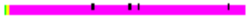
Q91XA2	Golgi membrane protein 1	Golm1	2	NaN	NaN	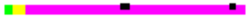
Q69ZN6	N-acetylglucosamine-1-phosphotransferase subunits alpha/beta	Gnptab	1	NaN	NaN	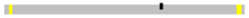
Q8K1B9	Polypeptide N-acetylgalactosaminyltransferase 18	Galntl4	2	NaN	NaN	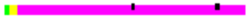
P97402	N-acetyllactosaminide beta-1,6-N-acetylglucosaminyl-transferase	Gcnt2	1	NaN	NaN	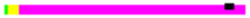
Q6P2L7	Protein CASC4	Casc4	3	NaN	NaN	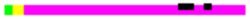
Q9Z222	N-acetyllactosaminide beta-1,3-N-acetylglucosaminyltransferase 2	B3gnt2	1	NaN	NaN	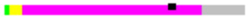

Suppl. Tab. 3: **Identified transmembrane type 3 proteins in BACE DKO and WT CSF** (UniProt subcellular location: Single-pass type III membrane protein [SL-9907])

UniProt AC	Protein names	Gene names	Unique peptides	Ratio (DKO/WT)	p-value	Topology (UniProt)
P41216	Long-chain-fatty-acid--CoA ligase 1	Acsl1	7	0.83	7.51E-01	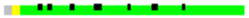
Q04646	Sodium/potassium-transporting ATPase subunit gamma	Fxyd2	2	0.93	7.46E-01	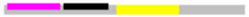

Suppl. Tab. 4: **Identified transmembrane type 4 proteins in BACE DKO and WT CSF** (UniProt subcellular location: Single-pass type I membrane protein [SL-9908])

UniProt AC	Protein names	Gene names	Unique peptides	Ratio (DKO/WT)	p-value	Topology (UniProt)
Q9QY76	Vesicle-associated membrane protein-associated protein B	Vapb	2	NaN	NaN	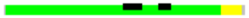

Suppl. Tab. 5: **Identified multi-pass transmembrane proteins in BACE DKO and WT CSF** (UniProt subcellular location: Multi-pass membrane protein [SL-9909])

UniProt AC	Protein names	Gene names	Unique peptides	Ratio (DKO/WT)	p-value	Topology (UniProt)
Q60932	Voltage-dependent anion-selective channel protein 1	Vdac1	14	0.49	1.30E-01	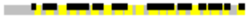
Q64518	Sarcoplasmic/endoplasmic reticulum calcium ATPase 3	Atp2a3	2	0.49	1.39E-01	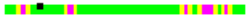
Q8R429	Sarcoplasmic/endoplasmic reticulum calcium ATPase 1	Atp2a1	37	0.49	9.49E-02	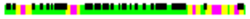
P48962	ADP/ATP translocase 1	Slc25a4	15	0.57	1.72E-01	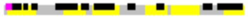
Q6PIE5	Sodium/potassium-transporting ATPase subunit alpha-2	Atp1a2	18	0.59	3.03E-01	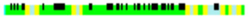
Q8BH59	Calcium-binding mitochondrial carrier protein Aralar1	Slc25a12	7	0.61	NaN	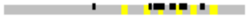
Q8K182	Complement component C8 alpha chain	C8a	30	0.65	5.93E-01	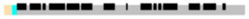
Q9DBG6	Dolichyl-diphosphooligosaccharide--protein glycosyltransferase subunit 2	Rpn2	2	0.66	NaN	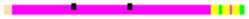
Q8VEM8	Phosphate carrier protein, mitochondrial	Slc25a3	8	0.70	4.69E-01	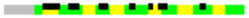
Q99P72	Reticulon-4	Rtn4	2	0.79	5.37E-01	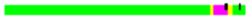
P00405	Cytochrome c oxidase subunit 2	Mtco2	2	0.85	6.57E-01	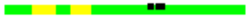
Q8C145	Zinc transporter ZIP6	Slc39a6	3	0.87	3.07E-01	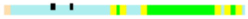
Q9QY42	Prosaposin receptor GPR37	Gpr37	6	0.93	6.69E-01	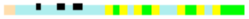
Q6PE13	Proline-rich transmembrane protein 3	Prrt3	4	0.96	7.76E-01	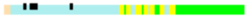
Q6P5F6	Zinc transporter ZIP10	Slc39a10	4	0.96	8.14E-01	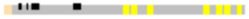
Q80TS3	Latrophilin-3	Lphn3	5	1.00	NaN	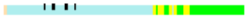
Q9R0M0	Cadherin EGF LAG seven-pass G-type receptor 2	Celsr2	8	1.01	9.80E-01	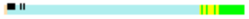
P06683	Complement component C9	C9	24	1.04	9.33E-01	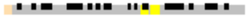
O70622	Reticulon-2	Rtn2	3	1.07	NaN	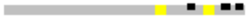
Q61735	Leukocyte surface antigen CD47	Cd47	2	1.08	NaN	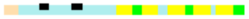
Q8VDN2	Sodium/potassium-transporting ATPase subunit alpha-1	Atp1a1	7	1.09	NaN	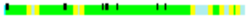
Q8K135	Dyslexia-associated protein KIAA0319-like protein	Kiaa0319l	4	1.10	6.67E-01	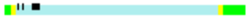
Q99JG2	Prosaposin receptor GPR37L1	Gpr37l1	4	1.11	4.40E-01	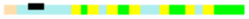
Q9Z0M6	CD97 antigen	Cd97	4	1.13	NaN	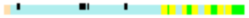
P35762	CD81 antigen	Cd81	2	1.14	4.95E-01	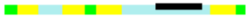
P04919	Band 3 anion transport protein	Slc4a1	22	1.15	8.83E-01	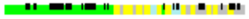
Q80TR1	Latrophilin-1	Lphn1	4	1.21	2.35E-01	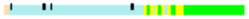
Q9Z2W8	Glutamate receptor 4	Gria4	9	1.29	2.70E-01	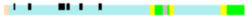
Q3UHD1	Brain-specific angiogenesis inhibitor 1	Bai1	4	1.34	1.83E-02	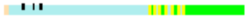
Q8CGM1	Brain-specific angiogenesis inhibitor 2	Bai2	7	1.43	1.32E-01	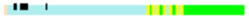
G5E8Q8		Gpr116	2	NaN	NaN	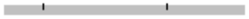

UniProt AC	Protein names	Gene names	Unique peptides	Ratio (DKO/WT)	p-value	Topology (UniProt)
Q8JZQ2	AFG3-like protein 2;AFG3-like protein 1	Afg3l2	2	NaN	NaN	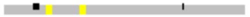
Q80ZF8	Brain-specific angiogenesis inhibitor 3	Bai3	2	NaN	NaN	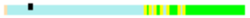
Q08857	Platelet glycoprotein 4	Cd36	2	NaN	NaN	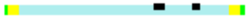
Q924X2	Carnitine O-palmitoyltransferase 1, muscle isoform	Cpt1b	4	NaN	NaN	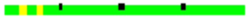
P56475	Gamma-aminobutyric acid receptor subunit rho-1	Gabrr1	1	NaN	NaN	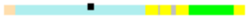
Q91VS7	Microsomal glutathione S-transferase 1	Mgst1	1	NaN	NaN	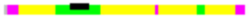
Q61885	Myelin-oligodendrocyte glycoprotein	Mog	1	NaN	NaN	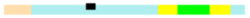
Q9D023	Mitochondrial pyruvate carrier 2	Mpc2	3	NaN	NaN	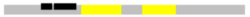
Q791V5	Mitochondrial carrier homolog 2	Mtch2	1	NaN	NaN	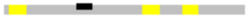
P03921	NADH-ubiquinone oxidoreductase chain 5	Mtnd5	1	NaN	NaN	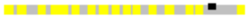
E9PZQ0	Ryanodine receptor 1	Ryr1	2	NaN	NaN	
Q9CXV1	Succinate dehydrogenase [ubiquinone] cytochrome b small subunit, mitochondrial	Sdhd	1	NaN	NaN	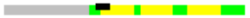
P53986	Monocarboxylate transporter 1	Slc16a1	1	NaN	NaN	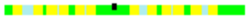
P56564	Excitatory amino acid transporter 1	Slc1a3	2	NaN	NaN	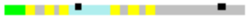
Q9CR62	Mitochondrial 2-oxoglutarate/malate carrier protein	Slc25a11	3	NaN	NaN	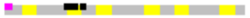
Q5I012	Putative sodium-coupled neutral amino acid transporter 10	Slc38a10	1	NaN	NaN	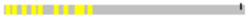
P31648	Sodium- and chloride-dependent GABA transporter 1	Slc6a1	1	NaN	NaN	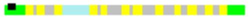
O89104	Synaptophysin-like protein 2	Sypl2	3	NaN	NaN	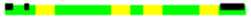
Q3TMP8	Trimeric intracellular cation channel type A	Tmem38a	1	NaN	NaN	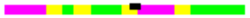
P12242	Mitochondrial brown fat uncoupling protein 1	Ucp1	3	NaN	NaN	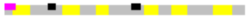
Q3UDR8	Protein YIPF3	Yipf3	1	NaN	NaN	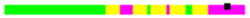

Suppl. Tab. 6: **Identified GPI-anchored proteins in BACE DKO and WT CSF** (UniProt subcellular location: GPI-anchor [SL-9902]). Proteins that are annotated as transmembrane type 1 proteins as well as GPI-anchored proteins are indicated with a yellow background.

UniProt AC	Protein names	Gene names	Unique peptides	Ratio (DKO/WT)	p-value	Topology (UniProt)
Q9QUR8	Semaphorin-7A	Sema7a	27	0.81	3.12E-01	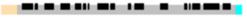
P10810	Monocyte differentiation antigen CD14	Cd14	11	0.82	4.60E-01	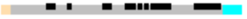
Q8R2G4	Ecto-ADP-ribosyltransferase 3	Art3	5	0.90	4.92E-01	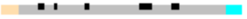
Q61468	Mesothelin;Megakaryocyte-potentiating factor	Msln	5	0.95	7.49E-01	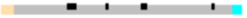
Q9D7F2	Ly6/PLAUR domain-containing protein 6B	Lypd6b	3	0.97	9.06E-01	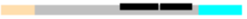
Q61330	Contactin-2	Cntn2	29	0.98	8.92E-01	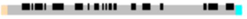
Q9WUC3	Lymphocyte antigen 6H	Ly6h	5	1.00	9.91E-01	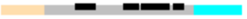
Q6PCX7	Repulsive guidance molecule A	Rgma	11	1.00	9.83E-01	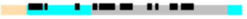
Q63912	Oligodendrocyte-myelin glycoprotein	Omg	7	1.02	9.27E-01	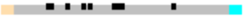
O88507	Ciliary neurotrophic factor receptor subunit alpha	Cntfr	4	1.03	8.56E-01	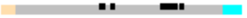
P13595	Neural cell adhesion molecule 1	Ncam1	24	1.03	8.81E-01	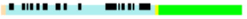
Q7M6Z0	Reticulon-4 receptor-like 2	Rtn4rl2	7	1.03	8.32E-01	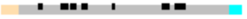
O08842	GDNF family receptor alpha-2	Gfra2	9	1.06	7.82E-01	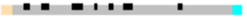
Q8BLC3	Ly6/PLAUR domain-containing protein 1	Lypd1	2	1.07	NaN	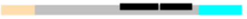
P01831	Thy-1 membrane glycoprotein	Thy1	5	1.08	7.07E-01	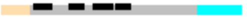
Q8CFV4	Neuritin	Nrn1	3	1.08	5.83E-01	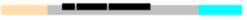
Q9QZF2	Glypican-1;Secreted glypican-1	Gpc1	14	1.09	6.39E-01	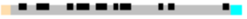
Q7TQ33	RGM domain family member B	Rgmb	4	1.09	7.18E-01	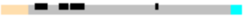
P12960	Contactin-1	Cntn1	29	1.10	6.08E-01	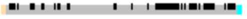
P29533	Vascular cell adhesion protein 1	Vcam1	13	1.12	5.22E-01	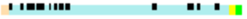
Q8BGN3	Ectonucleotide pyrophosphatase/phosphodiesterase family member 6	Enpp6	2	1.12	6.42E-01	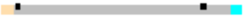
Q8BLK3	Limbic system-associated membrane protein	Lsamp	12	1.13	5.75E-01	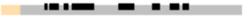
P04925	Major prion protein	Prnp	8	1.13	5.92E-01	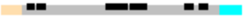
Q99PI8	Reticulon-4 receptor	Rtn4r	10	1.16	4.27E-01	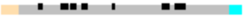
Q8R4G0	Netrin-G1	Ntng1	5	1.17	4.09E-01	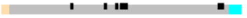
Q9R0S3	Matrix metalloproteinase-17	Mmp17	8	1.17	2.46E-01	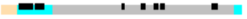
Q99PJ0	Neurotrimin	Ntm	8	1.19	4.28E-01	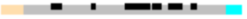
Q8CAL5	Glypican-5;Secreted glypican-5	Gpc5	5	1.19	2.87E-01	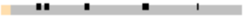
P0CW02	Lymphocyte antigen 6C1	Ly6c1	2	1.21	3.73E-01	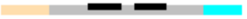
O35136	Neural cell adhesion molecule 2	Ncam2	5	1.21	3.95E-01	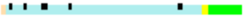

UniProt AC	Protein names	Gene names	Unique peptides	Ratio (DKO/WT)	p-value	Topology (UniProt)
Q8K0S5	Reticulon-4 receptor-like 1	Rtn4rl1	6	1.21	1.31E-01	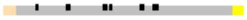
Q9WTR5	Cadherin-13	Cdh13	12	1.22	4.06E-01	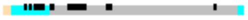
Q8R422	CD109 antigen	Cd109	5	1.23	4.33E-01	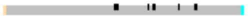
P21836	Acetylcholinesterase	Ache	12	1.26	3.42E-01	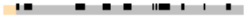
Q69Z26	Contactin-4	Cntn4	8	1.28	2.33E-01	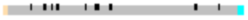
Q80Z24	Neuronal growth regulator 1	Negr1	10	1.32	2.34E-01	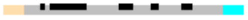
Q9JMB8	Contactin-6	Cntn6	10	1.39	1.54E-01	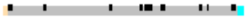
O55186	CD59A glycoprotein	Cd59a	2	1.57	1.49E-02	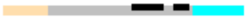
P52793	Ephrin-A1	Efna1	1	NaN	NaN	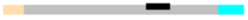
O08545	Ephrin-A3	Efna3	1	NaN	NaN	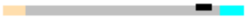
Q01721	Growth arrest-specific protein 1	Gas1	1	NaN	NaN	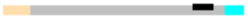
P97785	GDNF family receptor alpha-1	Gfra1	1	NaN	NaN	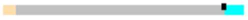
Q9R087	Glypican-6;Secreted glypican-6	Gpc6	1	NaN	NaN	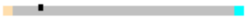
Q64253	Lymphocyte antigen 6E	Ly6e	1	NaN	NaN	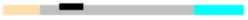
Q8C4W3	Neuritin-like protein	Nrn1l	3	NaN	NaN	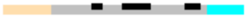
Q91X17	Uromodulin	Umod	3	NaN	NaN	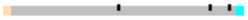
